# Cascading and pulse-like ruptures during the 2019 Ridgecrest earthquakes in the Eastern California Shear Zone

**DOI:** 10.1038/s41467-019-13750-w

**Published:** 2020-01-07

**Authors:** Kejie Chen, Jean-Philippe Avouac, Saif Aati, Chris Milliner, Fu Zheng, Chuang Shi

**Affiliations:** 1 Division of Geological and Planetary Sciences, California Institute of Technology, Pasadena, CA 91125 USA; 2 Department of Earth and Space Sciences, Southern University of Science and Technology, Shenzhen, 518055 Guangdong China; 3 Jet Propulsion Laboratory, California Institute of Technology, Pasadena, CA 91109 USA; 4 School of Electronic and Information Engineering, Beihang University, 37 Xueyuan Road, 100083 Beijing, China

**Keywords:** Natural hazards, Seismology

## Abstract

On July 4 2019, a M_w_ 6.5 earthquake, followed 34 h later by a M_w_ 7.1 event, struck Searles Valley, California. These events are part of a long-lived cluster of historical earthquakes along the Eastern California Shear Zone (ECSZ) which started in 1872 and are associated with temporarily elevated strain rates. We find that the M_w_ 6.5 event initiated on a right-lateral NW striking fault and then ruptured a left-lateral fault to the surface. This event triggered right-lateral slip during the M_w_ 7.1 earthquake. It started as a bilateral, crack-like rupture on a segment brought closer to failure by the M_w_ 6.5 event. The rupture evolved to pulse-like as it propagated at a relatively slow velocity (2 km/s) along a segment that was unloaded by the M_w_ 6.5 event. It stopped abruptly at the Coso volcanic area and at the Garlock Fault and brought some neighbouring faults closer to failure.

## Abstract (web summary)

The authors here investigate in the 2019 Ridgecrest earthquakes in Searles Valley, California. Based on geodetic and seismological data, the authors separate the event into two earthquakes (Mw 6.5 and 7.1) and produce kinematic slip models of the event.

## Introduction

**Fig. 1:**
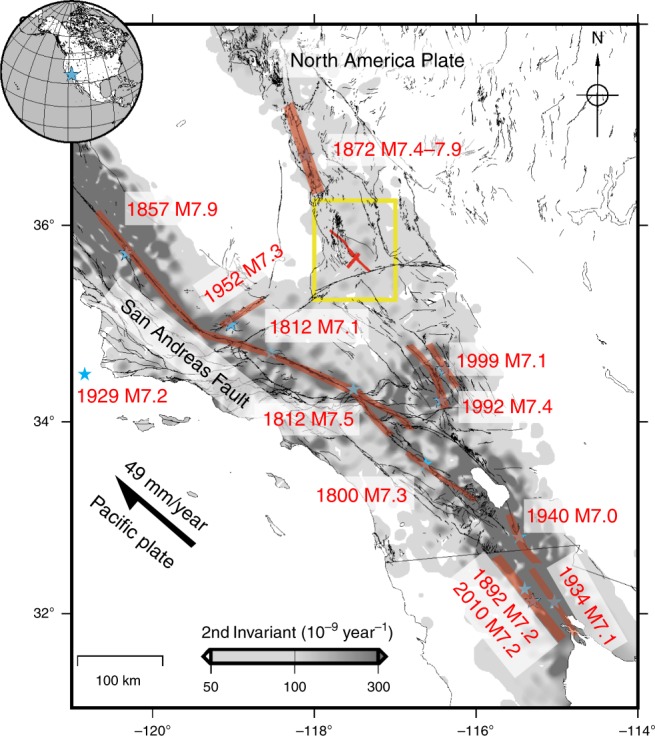
Setting of the 2019 Ridgecrest earthquakes. Strain rate over Southern California determined from geodetic measurements^50^ (grey shading). Active faults from the USGS database^51^ (https://earthquake.usgs.gov/hazards/qfaults/). Surface ruptures (red shading) of recent M > 7 earthquakes in Southern California since 1850 (https://earthquake.usgs.gov/hazards/qfaults/citation.php). The 1800 M7.3 rupture is from Salisbury et al^52^, the 1812 M7.1 rupture is from Lozos^53^, the 1812 M7.5 rupture is from Toppozada and Branum^54^, The 1892 M7.2 rupture is from Rockwell et al.^55^. Yellow box outlines the footprint of Fig. 2.

A sequence of earthquakes rattled Searles Valley near Ridgecrest, California, in July 2019. The two dominant events, a M_w_ 6.5 on July 4 followed 34 h later by a M_w_ 7.1 event on July 5, were felt over most of southern California and caused surface ruptures with minor damage to the nearby towns of Ridgecrest and Trona^1^. These earthquakes occurred within the Eastern California Shear Zone (ECZS), a zone of faulting and seismicity that runs east of the San Andreas Fault (SAF) system and joins with it in the Salton Trough (Fig. 1). The ECSZ includes the faults in Owens Valley along the eastern side of the Sierra Nevada^2^, and extends southward across the Mojave Desert^3,4^ (Fig. 1). The SAF system, including the San Jacinto fault and ECSZ, accommodate 80% of the 49 mm/yr of the northwestward motion of the Pacific Plate relative to stable North America. However the geodetic and geological data suggest significantly different rates across the ESCZ^5^. Global Positioning System (GPS) and other space based geodetic technique indicate consistently since the 1990s about 10 mm/yr of right-lateral shear^5–7^, while the geologic slip fault rates on the faults are typically of the order or 0.5–2 mm/yr and fall significantly short of matching the geodetic strain rate^3,5^. Conversely, the geodetic strain rate associated with some other faults, the left-lateral Garlock fault in particular, falls short of matching the Holocene geologic slip rate^7–9^.

The 2019 Ridgecrest earthquakes occurred within the exceptionally dense network of continuously recording GPS stations of the Plate Boundary Observatory^10^. Near-field high-rate (1 Hz) GPS records of large earthquakes are still rare and can provide unique insights into source characteristics^11,12^. Moreover the earthquakes occurred in a desert area that is particularly well suited for remote-sensing observations of ground deformation using radar^13^ or optical images^14^. The rich dataset available makes it not only possible to image the rupture process during the M_w_ 7.1 rupture and assess how this relates to the fault geometry, but also to study the possible effect of the static stress changes induced by the M_w_ 6.5 foreshock. It is indeed generally considered that the fault geometry and pre-existing stresses are primary factors controlling the nucleation, growth and arrest of seismic ruptures^15,16^.

In this study we therefore use remote sensing observations, high-rate GPS geodetic records and seismological waveforms to produce detailed source models of these earthquakes. We draw implications regarding the factors that influence the rupture process during these earthquakes and discuss how they relate to historical seismicity and geodetic strain in the ECSZ.

## Results

### Surface deformation measured from optical image correlation

**Fig. 2:**
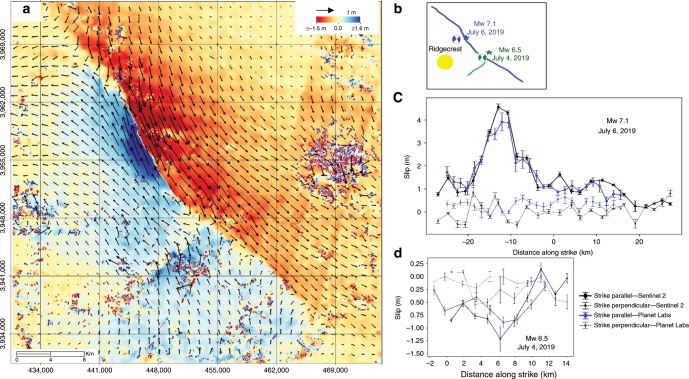
Surface deformation due to the 2019 Ridgecrest earthquakes measured from optical image correlation. **a** Surface displacement (arrows) and amplitude of NS component (shading) measured from correlation of Sentinel-2 images acquired on June 28 and July 08, 2019. **b** Simplified fault ruptures derived from the Sentinel-1 and Planet Labs data with GCMT focal mechanisms and epicentres of the M_w_ 7.1 and M_w_ 6.5 earthquakes from the USGS. **c** Strike-parallel (positive for right-lateral) and strike-perpendicular (positive in extension) component of surface fault slip measured from the Sentinel-1 and Planet Labs image. **d** Same as **c** for the M_w_ 6.5 earthquake.

We used optical image subpixel correlation to map the surface ruptures and fault slip. We selected publically-available Sentinel-2 and Planet Labs imagery with ground sample distance of 10 and 3 m, respectively. We used Sentinel-2 images acquired on 28 June and 8 July, so they were acquired before and after the two mainshocks, and Planet Labs images acquired on the 4 and 5 July at 11:11 PST, which allowed for distinguishing of the two events. These images were correlated to estimate the horizontal surface displacement field with a ground resolution on the order of a few hundred meters (Fig. 2). In practice the measurements are affected by various kinds of artefacts due to orthorectification errors, which might be due to inaccuracies of the digital elevation model used by the image providers, or to inaccurate geometric modelling of the images due to the satellite jitter or charge-coupled device (CCD) alignments^17^. Despite the artefacts, the surface ruptures are clearly visible in the offset maps measured from the Sentinel-2 and Planet Labs images (Fig. 2 and Supplementary Fig. 1). Both datasets clearly show one main rupture trending NW, which is visible only in the correlation of images spanning the M_w_ 7.1 event and a more minor rupture trending NE which is visible only in the correlation of images spanning the M_w_ 6.5. The measurements from the Sentinel-2 and Planet Labs images are very consistent and were used to map the fault trace for each event. The total fault slip, including the strike-slip and the strike-perpendicular components, are measured from profiles oriented perpendicular to the fault trace and estimated by linear extrapolation of the surface discontinuity across the observable fault-zone (see “Methods” section for details).

Although small geometrical fault-scale complexities can be identified, these measurements show that the M_w_ 7.1 event produce a nearly continuous fault trace over about 60 km, with an average of 1.60 ± 0.22 m of right-lateral surface slip (Fig. 2c). The strike-perpendicular component is very small within uncertainties (0.30 ± 0.15 m) so the slip vector is nearly everywhere parallel to the fault trace. The USGS W-phase moment tensor is consistent with these measurements (Fig. 2b).

Similarly, our measurements indicate that the M_w_ 6.5 event ruptured a ~15 km long fault striking NE with an average of 0.55 ± 0.08 m of left-lateral surface slip (Fig. 2d). The strike-perpendicular component is almost null with a small reverse component of 0.08 ± 0.08 m.

### Kinematic source models of the M_w_ 7.1 and M_w_ 6.5 earthquake

We determine a kinematic source model of the M_w_ 7.1 earthquake using the remote sensing data to constrain the fault geometry and the static displacements combined with the high-rate GPS and teleseismic records to constrain the time evolution of the rupture. The fault geometry is constrained from the surface ruptures (Fig. 2) and from the W-phase moment tensor which suggests that the fault dips 85° NE. We also use line of sight displacements measured from Interferometric Synthetic Aperture Radar (InSAR) provided by the Advanced Rapid Imaging and Analysis (ARIA) Project (https://aria.jpl.nasa.gov/) of the Jet Propulsion Laboratory (See “Methods” section and Supplementary Fig. 6 for image footprints and date of acquisition). We use the GPS records of ground displacement sampled at 1 Hz, along with selected waveforms with a good azimuth coverage from Incorporated Research Institutions for Seismology, and filtered them in the [0.005, 0.4] Hz frequency band (see “Methods” section and Supplementary Fig. 4). We determined a best fitting source model using a least squares procedure with a specified rupture expansion speed using the multiple time window approach^18^ (See “Methods” section and Supplementary Figures for more details). We additionally impose the surface fault slip measured from optical image correlation on the shallowest sub-fault patches. The SAR measurements show the total displacements due to the M_w_ 6.5 and M_w_ 7.1 earthquake. However, the model is probably not biased significantly because of the use of the high-rate GPS data and the fact that the fault geometry is constrained from the surface ruptures of only the M_w_ 7.1 event. The residuals clearly show a signal consistent with the effect of the M_w_ 6.5 earthquake (see Supplementary Fig. 8). We use these residuals to constrain the source model of the M_w_ 6.5 earthquake. We follow the same procedure for the M_w_ 7.1 event by constructing a fault model based on the surface ruptures and W-phase focal mechanism. We impose surface slip to match the measurements from optical image correlation and invert for the slip model that best fits the GPS and teleseismic waveforms as well as the InSAR residuals. The epicentral location and the early aftershocks suggest that the M6.5 event started on a right-lateral fault close to but east of the fault that ruptured in the M_w_ 7.1 event. Our source model therefore includes such a fault that didn’t break the surface. The best fitting solution fits well the static GPS data, and the remote sensing data, and we therefore consider that the slip distribution on the two nearly orthogonal faults is reasonably well constrained. The details of the rupture kinematics are not well resolved as suggested by the misfit between the predicted and measured GPS waveforms.

## Discussion

**Fig. 3:**
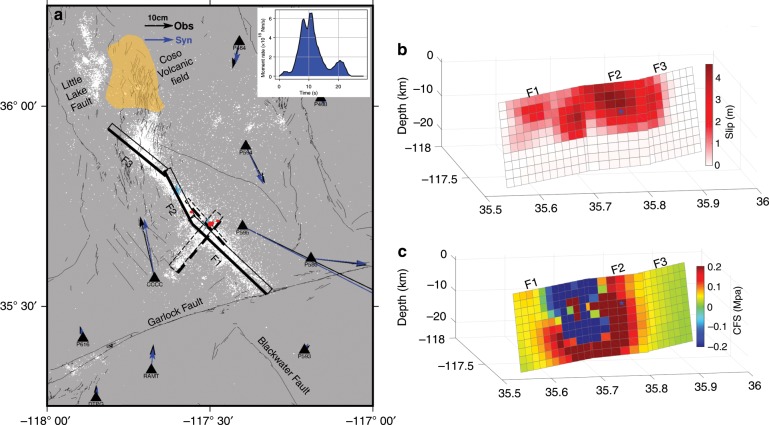
Source model of the M_w_ 7.1 earthquake of July 6, 2019. **a** Co-seismic GPS measurements (black arrow) and synthetics (blue arrow). White dots show seismicity after the M_w_ 6.5 event until 1 August, 2019, red dots are foreshocks. The catalogue is from the Southern California Seismic Network which is available on-line (https://scedc.caltech.edu/eq-catalogs/index.html). Solid and dashed rectangle outlines projections of the faults adopted in this study for the M_w_ 7.1 and M_w_ 6.5 events, respectively, and the thick edges are at the surface. Left top corner, moment rate function derived. **b** Model of the slip distribution for fault F1, F2 and F3, the blue star shows the hypocenter location. **c** Coulomb Stress Failure on the fault of the M_w_ 7.1 event induced by the M_w_ 6.5 earthquake, note that the color bar is saturated.

**Fig. 4:**
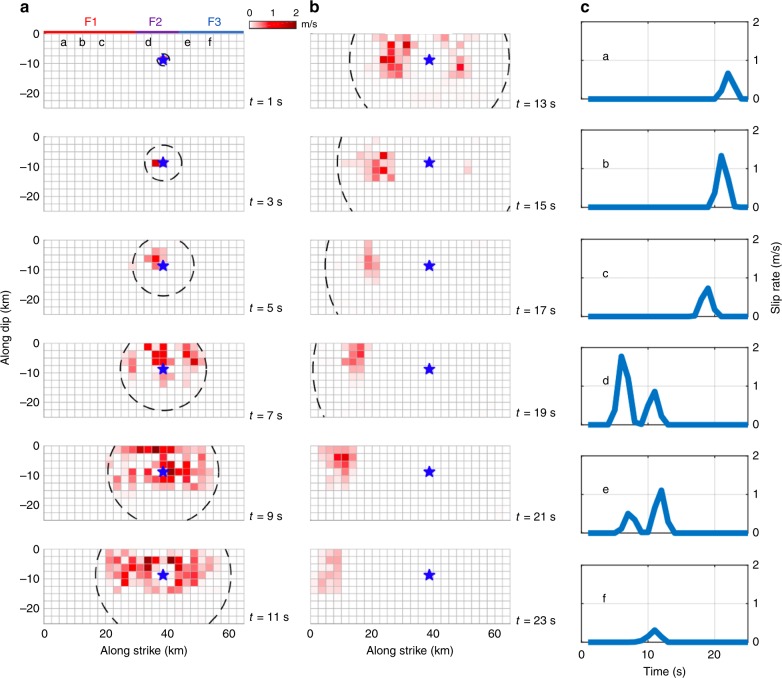
Time evolution of the rupture during the M_w_ 7.1 Ridgecrest earthquake. **a**, **b** Snapshots of the rupture expansion history every two seconds. Colours shading indicates the fault slip rate average over 2 s intervals. The black dashed contour presents the pseudo-rupture front with a rupture velocity of 2.0 km/s. F1, F2, F3 correspond to the faults in Fig. 3. **c** Presents slip rate at the six locations in **a**. Time is relative to the hypocentral origin.

The earthquake catalogue of the Southern California Seismic Network shows that the Ridgecrest sequence started with a sequence of foreshocks which were detected within 10 km of the epicenter of the M_w_ 7.1 event (Fig. 3a). The largest earthquake preceding the M_w_ 6.5 foreshock was a M_w_ 4.0 earthquake 30 min earlier. According to our model (see Fig. S6), the M_w_ 6.5 earthquake itself is a compound event which initiated on the right-lateral NW striking fault, released half of the moment, and then activated the left-lateral fault. It released a total moment of 7.43 × 10^18^ N·m (equivalent to M_w_ 6.5) in about 10 s. Earthquakes with magnitude up to M_w_ 5.4 swarmed for 34 h around the two fault planes that were activated during the M_w_ 6.5. Then, on July 5, one event was able to grow bigger and evolved into the M_w_ 7.1 mainshock. Our kinematic source model shows that, during this event, a total moment of 4.85 × 10^19^ N·m (M_w_ 7.06) was released in about 23 s (Fig. 3a). The rupture initiated on segment F2 (striking N–NW), a fault segment that was brought closer to failure by the M_w_ 6.5 event (Fig. 3c). We estimate the static Coulomb stress increase^19^ at the location of the hypocenter to have been 0.2 MPa. The rupture expanded as a crack up-dip and bilaterally during the first 11 s (Fig. 4 and Supplementary Movie 1), leading to up to ~5 m of slip on segment F2 (Fig. 3b). From then on, it expanded as a narrow slip-pulse that propagated southeastward on segment F1 and northwestward on segment F3. The pulse died rapidly along the northernmost segment F3 as the rupture approached the Coso volcanic area. However, it was able to propagate over 30 km along the southernmost segment F1 until its termination at the intersection with the Garlock fault. The rupture front expanded throughout the rupture process at a rate of 2 km/s (see “Methods” section and Supplementary Fig. 11 for the determination of the rupture velocity). The slip velocity reached a maximum of about 2 m/s on segment F2. The width of the pulse is estimated to have been 5 km and was associated with a peak slip velocity 0.5–1 m/s.

It should be noted that the source parameterization adopted in this study does not enforce a pulse-like rupture. As a result, the transition to pulse-like rupture after ~11 s into the rupture process is a remarkable feature of this earthquake which is required by the high-rate GPS and seismological records. Pulse-like ruptures have long been inferred from seismological observations^20^ and have now been documented repeatedly in particular thanks to the increasing availability of near-field high-rate GPS records^12^. The reasons for pulse-like ruptures are still debated. They might reflect geometric effects but seem to require, in any case, strong velocity weakening friction on the fault^21^. The initial stress can be a factor too: both theoretical and experimental studies show a transition from pulse-like to crack-like ruptures occurs as the pre-stress increases^22,23^. This factor could have played a role during the M_w_ 7.1 event. It is indeed intriguing that the rupture initiated and expanded as a crack on F2 where the static Coulomb stress increased substantially due to the M_w_ 6.5 event, but propagated as a pulse on the segment where the stress level decreased (F1) or didn’t increase much (F3) (Fig. 3c). The stress variations due to the M_w_ 6.5 events are, however, small compared with the stress drop during the M_w_ 7.1 event, which is estimated to have an average of ~10 MPa using the energy-based approach of Noda et al.^24^. It is possible that due to their orientation, F1 and F3, which both strike NW (313°and 305°, respectively), were initially both under lower Coulomb stress than F2, which strikes NNW (333°).

The M_w_ 7.1 rupture reached close to the intersection of F1 with the Garlock Fault suggesting a geometric control at its southern termination. To the north, the rupture arrested close to the Coso volcanic area where a zone of high heat flow encompasses an area containing numerous Pleistocene volcanic vents and domes^25^ (Fig. 3a). The northern termination of the M_w_ 7.1 event is reminiscent of the arrest of the Kumamoto earthquake close to the Aso volcanic system^26^. High pore pressure due to the hydrothermal activity and higher crustal temperatures may have favoured fault creep at shallow depth. The resulting reduced stress level could then have inhibited the propagation of the rupture during the M_w_7.1 event. It could have also inhibited aftershocks, as was also observed at Aso volcano following the Kumamoto earthquake^26^, as well as remotely triggered earthquakes^27^.

Interestingly, the 2019 Ridgecrest earthquakes seem to be part of a cluster of large events concentrated along the ECSZ. The cluster includes the 1992, M_w_ 7.3 Landers and the 1999, M_w_ 7.1 Hector Mine earthquakes in the Mojave Desert (Fig. 1). A few M > 6 earthquakes had additionally occurred earlier in the area, namely the M6.5 Manix Lake earthquake, and two more minor (~M6) events in 1975 and 1979. Farther north, the ECSZ produced a major earthquake in 1872 with right-lateral ruptures over a distance of about 120 km^28^, which is estimated to have been as large as M_w_ 7.8–7.9 and was one of the largest historical events in California^29^. These events are close in time given that most faults in the ECSZ (with exception of the Garlock fault) that have been studied for its paleoseismology exhibit a recurrence interval of 6–10 ka or longer. This clustering at the regional scale is somewhat surprising as geodetic strain rates within the ECSZ accounts for only about 20% of the relative plate motion, compared with more than 60% on the rest of the SAF system. The fact that the geodetic rate exceeds geological rates suggests however that the cluster is possibly associated with an on-going pulse of geodetic strain rates^4,30,31^.

The remote-sensing, seismological and geodetic data assembled in this study make it possible to describe the details of how the 2019, Ridgecrest earthquake sequence unfolded. It reveals a sequence of triggering, where small foreshocks initiated on a secondary fault which produced a complex rupture of two conjugate faults on July 4, leading to the eventual triggering of the M_w_ 7.1 event on July 5. The larger event was possibly arrested due to a combination of geometric (at the intersection with the Garlock fault), and probably hydromechanical (at Coso) effects. The rupture evolved from a crack-like to a pulse-like rupture. This evolution correlates with strike changes and the co-seismic stress changes due to the M_w_ 6.5 event. These features are noteworthy as they are qualitatively consistent with theory in principle, but further investigations based on dynamical simulations will be needed for a more quantitative test.

With regard to seismic hazard, the Ridgecrest sequence belongs to a late Holocene and historical cluster of seismicity in the ECZS, which includes the 1872 Owens Valley earthquake and the more recent 1947 Manix Lake, 1992 Landers, and 1999 Hector Mine events, all of which are associated with a zone of elevated strain rate measured from geodesy and remote sensing^5–7^. The high-strain rate is probably transient in view of the lower rate derived from geological studies^3^ and might explain the regional clustering of large magnitude earthquakes that seem to characterize this area^4,30,31^ and which is not taken into account in seismic hazard studies. A number of faults adjacent to the Ridgecrest ruptures that belong to the same zone of elevated high strain, and have not ruptured recently, must have been brought closer to failure. They include the Little Lake Fault, which connects the northern end of the recent M_w_ 7.1 rupture with the Owens Valley Fault^32^, and several right-lateral faults south of the Garlock Fault, including in particular the Blackwater Fault^3^. The Garlock fault itself was unclamped west of its intersection with the M_w_ 7.1 rupture. Further earthquakes rupturing some of these faults in the future would be a natural continuation of the historical cluster although we admit that the significance and cause of this cluster are open questions.

## Methods

### Optical image correlation of Sentinel-2 and Planet Labs images

In order to assess the horizontal surface displacement induced by the two earthquakes (M_w_ 6.5 and M_w_ 7.1), we applied subpixel image correlation using the COSI-Corr technique^33^. Two Copernicus Sentinel-2 orthorectified-optical imagery covering the Ridgecrest region were selected before and after the two earthquakes. Pre and post-earthquake images were acquired, respectively, on June 28, 2019 (7 days before the M_w_ 6.5 earthquake) and on July 8, 2019 (3 days after the M_w_ 7.1 earthquake).

Sentinel-2 has a ground sampling distance of 10 m and is available in four different wavelengths (band 2-Blue, band 3-Green, band 4-Red and band 8-NIR). We correlated each band using the phase correlator of COSI-Corr which can in principle yield an accuracy of 1/50 pixel. A sliding multiscale window was used iteratively in two steps, a size window of 64 × 64 pixels (640 × 640 m^2^) was chosen for the first step of the correlation, before iterating the correlation with a window of 32 × 32 pixels (320 × 320 m^2^) for the second step. Because of the shape of the correlation kernel the ground resolution is about half the size of the sliding window. We set a sliding step of 4-pixels. The corresponding ground sampling distance is thus 40 m but the resolution is of the order of 150 m. For mitigating the effects of the high frequency noise on the accuracy of the correlation results, we set the frequency mask threshold as 0.9 and robust iteration as 2.

Each correlation leads to displacement maps in the E–W and N–S directions. The signal to noise ratio on offset measurements is determined from the normalized cross spectrum. The 1-σ uncertainty is ~20 cm. The measurements show strong striping artefacts typical of jitter effects and CCD misalignments. In principle these artefacts can be modelled and mitigated during the orthorectification procedure but this was not possible as the raw images are not distributed by European Space Agency (ESA). Some orthorectification errors due to the DEM inaccuracies are also visible. We therefore mitigated these various artefacts with post-processing.

Misalignment of CCD arrays produces stripes in the along-track direction and are common obvious artefacts of pushbroom systems. These artefacts were removed by subtracting the average along-track value from each column in the along-track geometry. A second de-stripping was carried out to deal with the across-track stripes due to attitude oscillations of the satellite (“jitter”). De-trending step was carried out to the correlation maps by removing a linear ramp, determined from stable areas far from the fault rupture, in order to discard errors caused by orthorectification errors.

To separate the surface, the deformation between the two earthquakes that are closely spaced in time, by ~34 h, we use 3 m resolution Planet Scope imagery (see Supplementary Fig. 2) acquired by Planet Labs. We use the image correlation approach to measure the horizontal surface deformation with a window size of 32 × 32 pixels and step of 9, and then apply a median filter (3 × 3 pixels) after correlation, which provides displacement at ~15 cm precision (1-σ). To measure the surface offset we use the COSI-Corr profiles orientated perpendicular to the fault trace with 8 km length and ~1 km width, and extrapolate the linear regression fit to either side of the fault to estimate the total fault offset over the fault-zone.

### Processing of high-rate GPS data

We processed 1-Hz continuous GPS data (see station distribution in Fig. S3a) from the Plate Boundary Observatory GNSS/GPS network for the day of the two events, respectively. GPS satellite orbits, Earth rotation parameters and 1-Hz GPS satellite clocks were estimated using Positioning And Navigation Data Analysis software following the strategies described in Zhang et al.^34^. Precise point positioning^35^ is then employed to estimate epoch-wise positions together with receiver clock errors, zenith troposphere delays and phase ambiguities. Specifically, we assume no constraints between neighbouring epochs, receiver clocks are treated as white noise while troposphere delays as random-walk parameters with a process noise of (1 mm/s)^1/2^. In addition, tidal displacements (solid Earth tide, pole tide and ocean tide loading) are modelled according to IERS Conventions 2010^36^. Antenna phase center offsets and variations are also corrected. All position estimates are aligned to the International Terrestrial Reference Frame (ITRF2008) and transformed into a local north, east, up frame.

### SAR interferometric data

We obtained three radar images from the Advanced Land Observing Satellite 2 (ALOS-2) operated by the Japan Aerospace Exploration Agency and the Copernicus Sentinel-1 satellite operated by the ESA. The interferograms were processed by the JPL-Caltech ARIA team, which have been archived at https://aria-share.jpl.nasa.gov/20190704–0705-Searles_Valley_CA_EQs/Interferograms/. The Sentinel-1 TOPS data were processed using InSAR Scientific Commuting Environment^37^. To form an interferogram with the shortest time span with the available acquisitions, the ALOS-2 interferogram is formed with ScanSAR-stripmap interferometry using a multi-mode InSAR processor added to ISCE^38,39^. Spatial and temporal coverage of SAR data are summarized in Supplementary Fig. 3.

### Data preprocessing

We only use static GPS offsets within 100 km of the epicenter. The high-rate GPS displacement waveforms were low-pass filtered to 0.4 Hz. Specifically, for the July 4 M_w_ 6.5 event, we find the signal-to-noise level of the vertical components of the high-rate displacement waveforms is so low that we exclude them for inversion. To reduce the computational burden, we subsampled the InSAR data using the quad tree technique^40^ and a total of 2363 InSAR line of sight points were included for the joint inversion (see Supplementary Fig. 8). We use the residuals of the ALOS-2 (see Supplementary Fig. 11a) from July 6 M_w_ 7.1 to constrain the inversion of the July 4 M_w_ 6.5 event.

We downloaded 35 teleseismic P ground recordings (see station distribution in Supplementary Fig. 9a) with 30° to 90° epicentral distances from IRIS. Instrument responses were deconvolved to retrieve displacement waveforms, which were then filtered with a bandpass filter at a [0.005, 0.4] Hz corner frequency and decimated to 1 Hz. We extracted 120-s-long samplings from the raw data, starting 6 s prior to the clearest first arrival of the P waves. We manually aligned P wave initial motions to the theoretical arrival time predicted by velocity model of preliminary reference earth model (PREM)^41^.

### Co-seismic rupture inversion

We perform a joint inversion based on ALOS-2 and Sentinel-1 measurements, static GPS offsets, 1-Hz GPS and broadband displacement waveforms using a multi-window code called Mudpy, which was originally developed by Melgar and Bock^42^.

For the M_w_ 7.1 event, the fault geometry was constrained by the trace of the rupture at the surface, which implied a varying strike along the fault. To that end, we use three fault planes to represent the rupture geometries. For the M_w_ 6.5 event, in addition to the NE trending rupture that is recovered by the optical images, distribution of the aftershocks implies that it should have probably ruptured a conjugate fault, and we adopt two fault planes to model this event. Geometries of the two fault planes are determined based on the surface rupture trace and W-phase moment tensor solutions. In terms of dip and rake angles, we assign their values based on global centroid moment tensor solutions^43^. All of the fault planes are further parameterized by a number of sub-faults along strike and dip, the detailed fault geometries are listed in Table S1.

The frequency-wavenumber integration method^44^ is adopted to compute Green’s functions for every near-field data type (static GPS, InSAR, 1-Hz GPS displacement waveforms) using a 1-D layered velocity proposed by Mori and Helmberger^45^ as shown in Table S2. The teleseismic Green’s functions are generated by propagator matrix approach^46^, velocity model by Mori and Helmberger^45^ is used for the source side and PREM^41^ is used for the receiver side. Besides, the same bandpass filter used for the waveforms is applied to the Green functions.

For each sub-fault, two vectors (see the vector rake angle in Table S1) are used and non-negative least square inversion is employed to account for the rake-varying slip. To recover the complexity of the sub-fault source time functions, we use 5 symmetric triangles with 3 s half-durations staggered by 1.5 s each for each sub-fault. To ensure a stability of the inversion result, we employ the first-order Laplacian regularization^18^. To calibrate the rupture speed in the inversion, we vary it between 1.2 km/s and 3.4 km/ in 0.2 km/s bins, and the variance reduction as a function of rupture speed are plotted in Supplementary Fig. 4. We conclude that all the data are best explained by a maximum rupture speed of 1.8–2.0 km/s for the M_w_ 7.1 event and 2.6 km/s for the M_w_ 6.5 event.

Relative weighting always presents a tricky issue for joint inversion^47^. In this study, each kind of data is first normalized by their own norm and then different weighting factors are tested (see Supplementary Fig. 5), and we find that an equal weighting among GPS, InSAR and P waves can fit all datasets well.

For the joint inversion, we ran 25 iterations with various spatial and temporal smoothing levels and chose the favored one based on Akaike’s Bayesian Information Criterion^48^, ABIC, against different smoothing factors as depicted in Supplementary Fig. 6. See comparison of observations and model predictions for the preferred slip model of M_w_ 7.1 event in Supplementary Figs. 7–9. Supplementary Figs. 10 and 11 summarize the results of the M_w_ 6.5 event.

To investigate the resolution of inversion, we performed a standard checkerboard test, in which a synthetic checkerboard slip distribution (Supplementary Fig. 12) was built, and all the data used for the inversion were simulated. The checkerboard test shows that for the M_w_ 7.1 event, even the deep slip can be reliably recovered. For the M_w_ 6.5 event, however, a poor deep slip resolution is found due to a relatively sparse dataset.

In order to further estimate uncertainties of the inverted slips, we conducted a jackknife test, through which 20% of the data were randomly removed each time and inversion on the reduced datasets was repeated for 100 times. The standard deviation together with the coefficient of variation (the standard deviation divided by the slip) for the two events are shown on Supplementary Fig. 13. While the absolute variability of slip on each patch is indicated by the standard deviation, the coefficient of variation can be a better measure for showing which features are persistent.

### Coulomb failure stress

Coulomb stress failure changes (ΔCFS) on a plane can be described as^49^:

$${\it{\mathrm\Delta }}\mathrm{CFS} = \tau _\beta - \mu ^\prime ({{\Delta }}\sigma _n),$$

where $\tau _\beta$ and ${\it{\Delta }}\sigma _n$ denote the shear and normal stress change, *μ′* is the coefficient of effective friction. We analyze ΔCFS due to the M_w_ 6.5 event on the rupture fault planes of the M_w_ 7.1 event. Considering the focal mechanism of the M_w_ 7.1 event, we assume a uniform rake angle of 180° for all of the three faults, the effective friction coefficient is set as 0.3, and the shear module is set at 40 GPa.

## Supplementary information

Supplementary Information

Peer Review

Description of Additional Supplementary Files

Supplementary Movie 1

## Data Availability

The Sentinel-2 and Planet Labs images are stored at 10.5281/zenodo.3542274 and 10.5281/zenodo.3542132, respectively. 1-Hz raw GPS observations are achieved by UNAVCO and publicly available at ftp://data-out.unavco.org/pub/highrate/1-Hz/rinex/2019/185 and ftp://data-out.unavco.org/pub/highrate/1-Hz/rinex/2019/187. Static co-seismic GPS offsets are provided by the Nevada Geodetic Laboratory (http://geodesy.unr.edu/). The interferograms were generated from Sentinel SAR images from ESA and Japanese Aerospace Agency Advanced Land Observing Satellite 2, which were distributed by the ARIA team at the Jet propulsion Laboratory (https://aria-share.jpl.nasa.gov/20190704–0705-Searles_Valley_CA_EQs/Interferograms/). The broadband teleseismic seismograms are publicly available from Incorporated Research Institutions for Seismology (http://ds.iris.edu/wilber3/find_stations/11058875). The earthquake catalogues are obtained from the Southern California Earthquake Center at Caltech (http://service.scedc.caltech.edu/eq-catalogs/date_mag_loc.php). The focal mechanisms plotted in Fig. 2b can be accessed at Global Centroid Moment Tensor catalogue (https://www.globalcmt.org/CMTsearch.html). All other data can be obtained from the lead author upon reasonable request (kjchen@caltech.edu).

## Code availability

The Mudpy software package to invert the co-seismic slip distribution is available at https://github.com/dmelgarm/MudPy, the COSI-Corr software package is for free download from http://www.tectonics.caltech.edu/slip_history/spot_coseis/. The various scripts for Coulomb stress change calculation, data analysis and plotting are available from the authors upon request.
