# Supplementary material for: Cascading and pulse-like ruptures during the 2019 Ridgecrest earthquakes in the Eastern California Shear Zone
Source: Nat Commun. 2020 Jan 7;11:22. doi: 10.1038/s41467-019-13750-w (PMC6946662; doi:10.1038/s41467-019-13750-w)
Supplement: Supplementary file 1 — Supplementary Information [file 41467_2019_13750_MOESM1_ESM.pdf]

Supplementary Information for

Cascading and pulse-like ruptures during the 2019 Ridgecrest earthquakes in Eastern California Shear Zone

by Chen et al.

Supplementary Figures 1 to 13

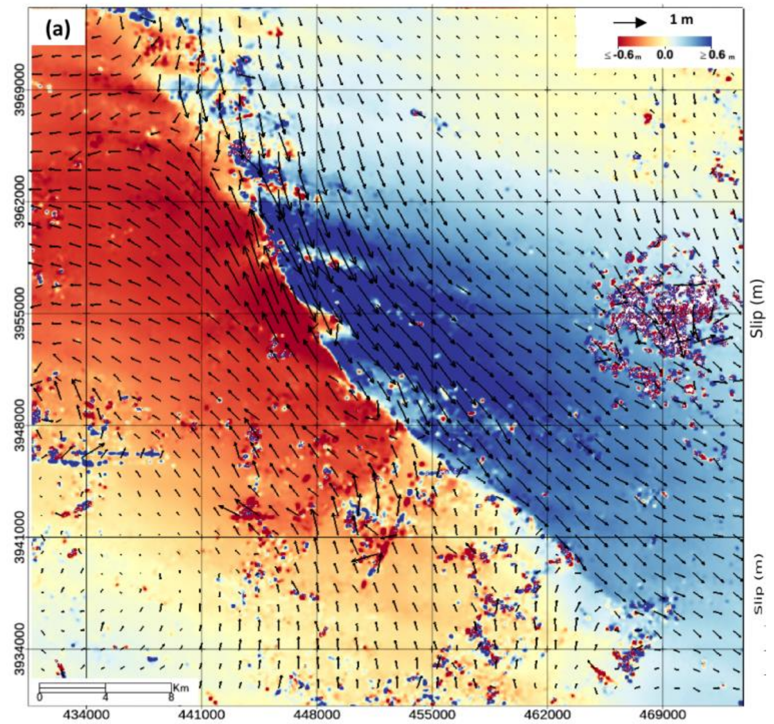

Supplementary Figure 1: Surface deformation due to the 2019 Ridgecrest earthquakes measured from optical image correlation. Surface displacement (arrows) and amplitude of EW component (shading) measured from correlation of Sentinel-2 images acquired on June 28 and July 08, 2019.

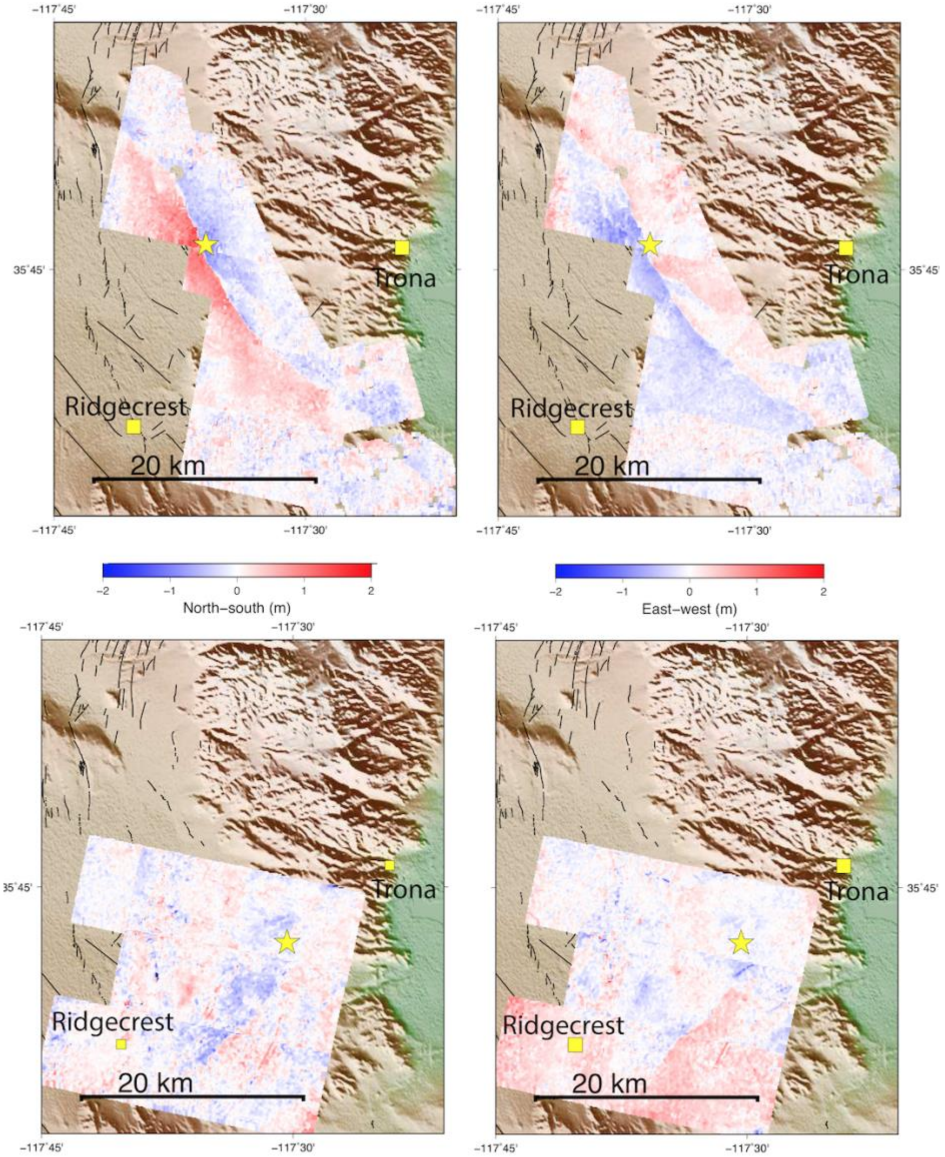

Supplementary Figure 2: **Optical correlation of Planet Scope imagery.** Top row shows surface deformation due to the Mw 7.1 earthquake, and bottom for the Mw 6.4. Left column shows north-south surface motion, right column shows east-west. The imagery was acquired daily, i.e., on July 6 and July 4, 2019, respectively.

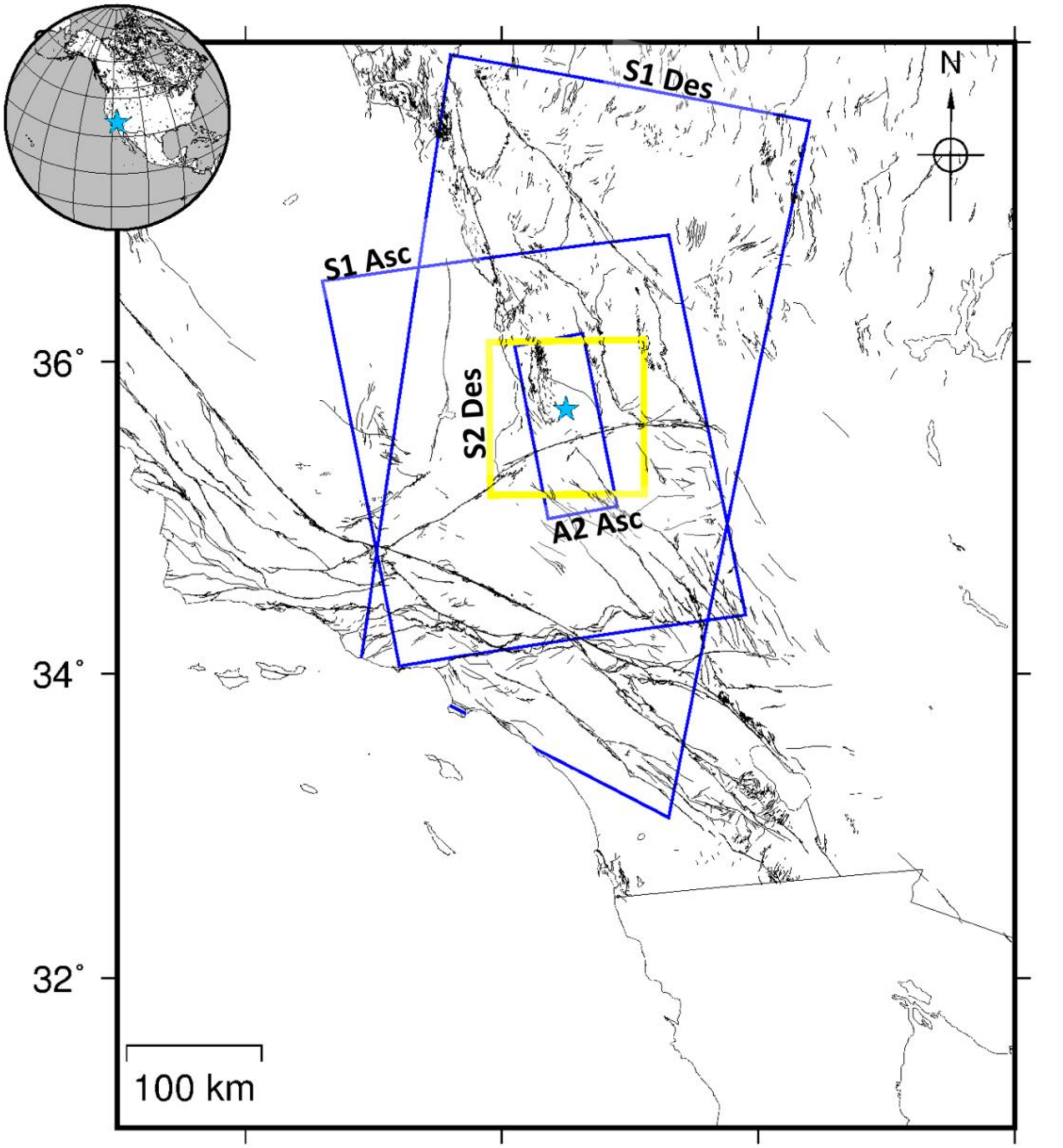

Supplementary Figure 3: Footprint of the optical and InSAR measurements used in this study. Yellow box outlines the footprint of the image correlation results from Sentinel-2 optical images of June 28 and July 08, 2019. The blue boxes outline the footprints of SAR data from ALOS-2 ascending (from April 16, 2018 to July 8, 2019), Sentinel-1 ascending (from July 4, 2019 to July 10, 2019) and descending (from July 4, 2019 to July 16, 2019) orbits.

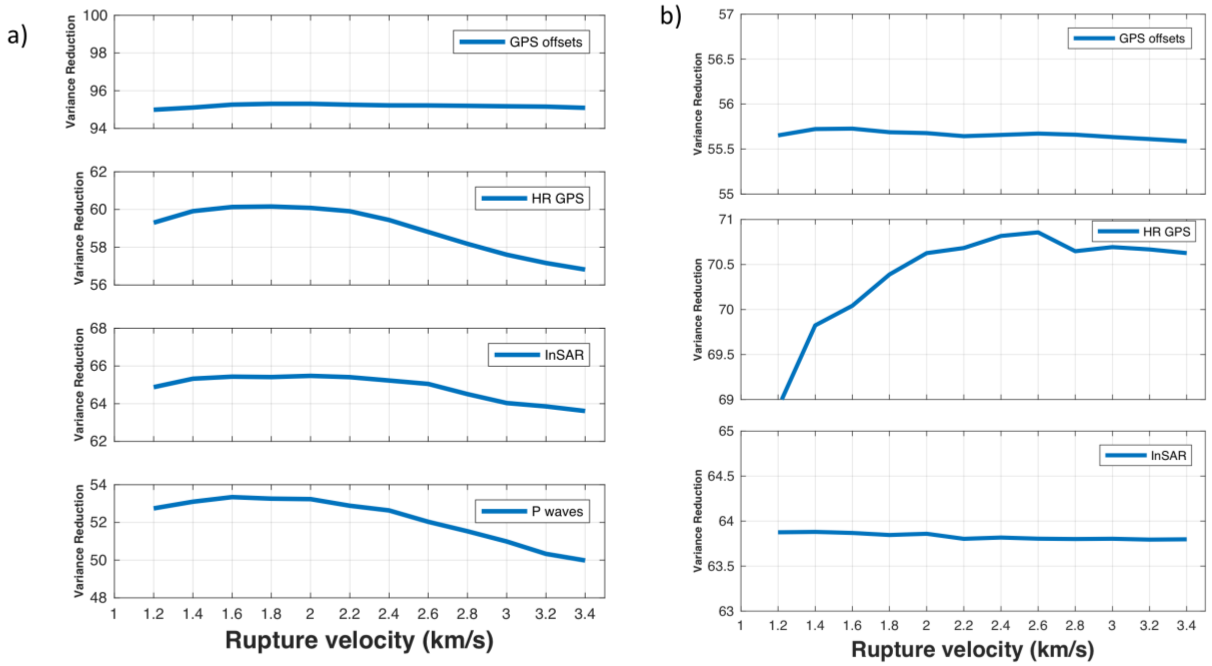

Supplementary Figure 4: Variance reduction to each kind of datasets as a function of maximum allowed rupture speed for the July 6 M_w 7.1 event a) and July 4 M_w 6.5 event b).

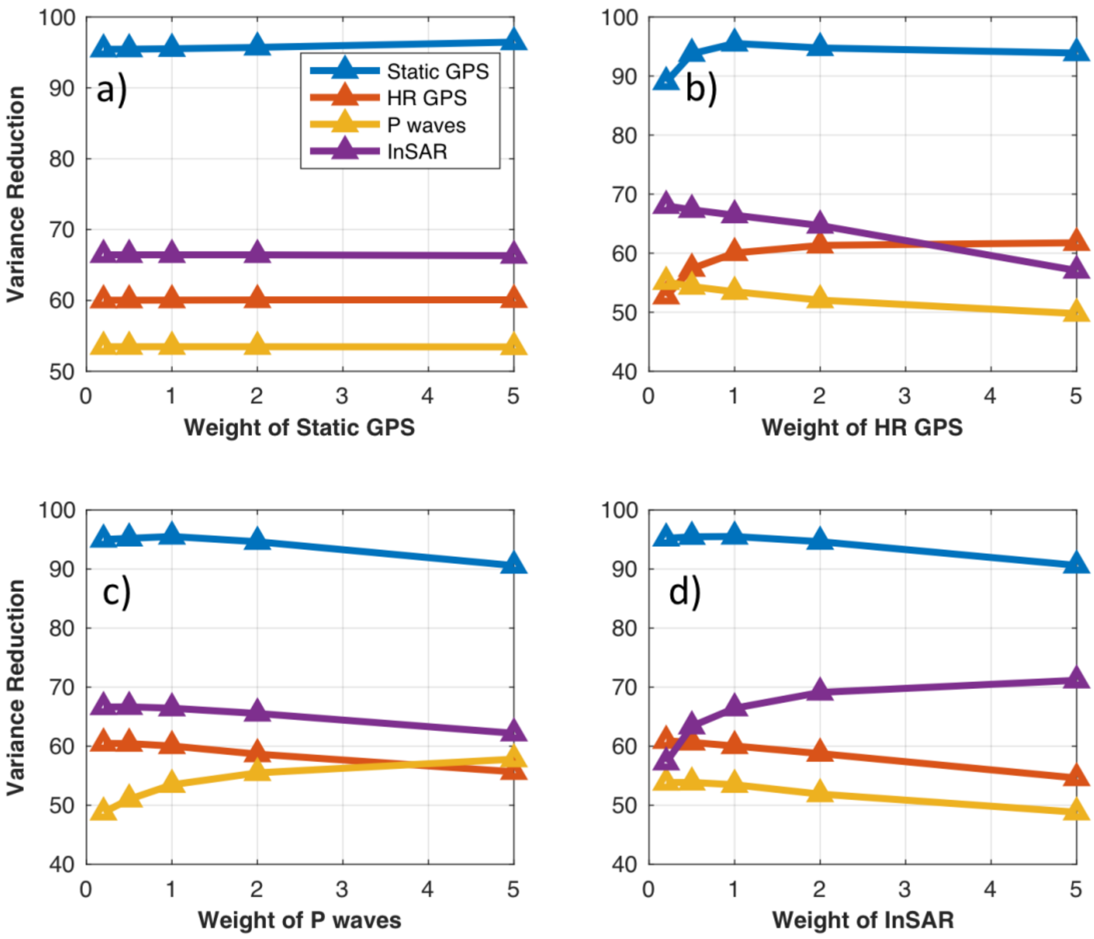

Supplementary Figure 5: Variance reduction to each kind of datasets with different weighting assignments to a) static GPS, b) high-rate GPS waveforms, c) P waveforms and d) InSAR measurements. For each test, we vary the weighting on only one dataset and keep the weighting assigned to the three other datasets to 1.

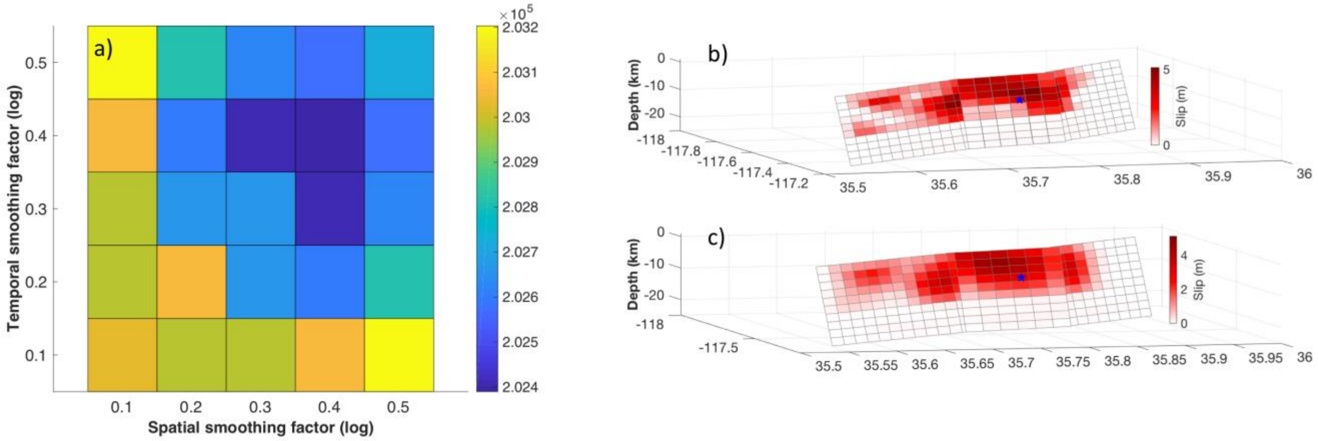

Supplementary Figure 6: a) Akaike's Bayesian Information Criterion against varying spatial and temporal smoothing levels. b) and c) are slip distribution with the weakest and strongest smoothing constrains for the Mw 7.1 event, respectively.

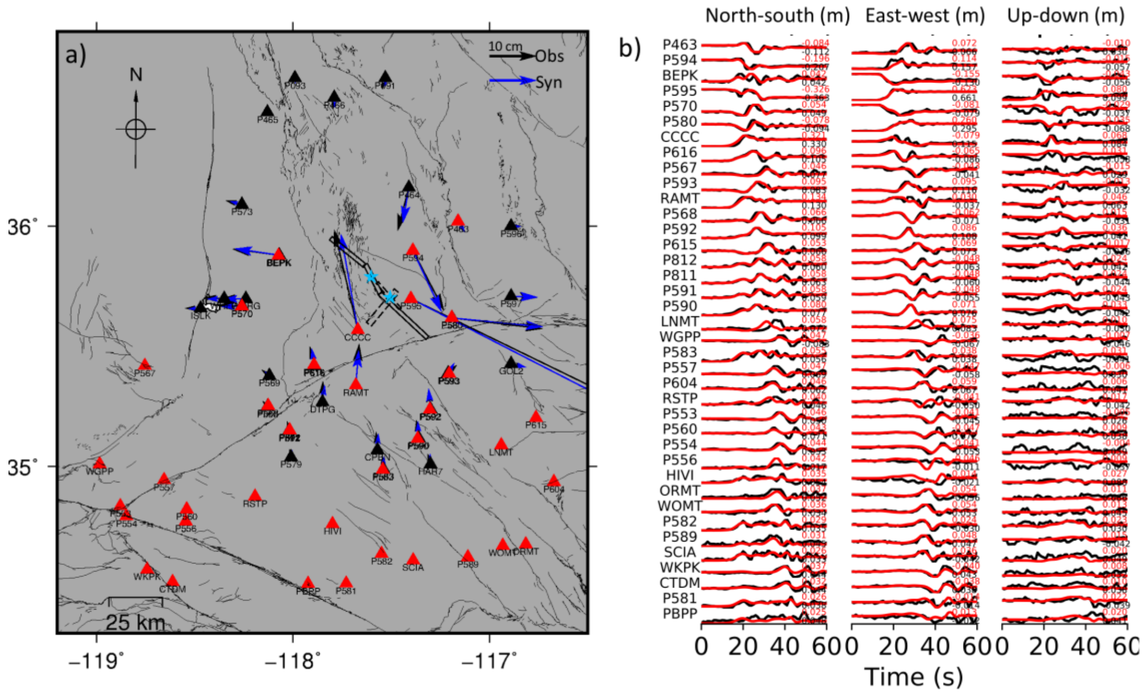

Supplementary Figure 7: GPS data fits for the 6 July M_w 7.1 event. a) Co-seismic GPS observations (black arrow) and fits (blue arrow). Solid and dashed rectangles outline faults geometries adopted to model the M_w 7.1 and M_w 6.5 events respectively. Red triangles denote the locations of high-rate GPS stations. b) High-rate GPS observations (black) and fits (red). The numbers at right indicate the maximum amplitude values for each waveform.

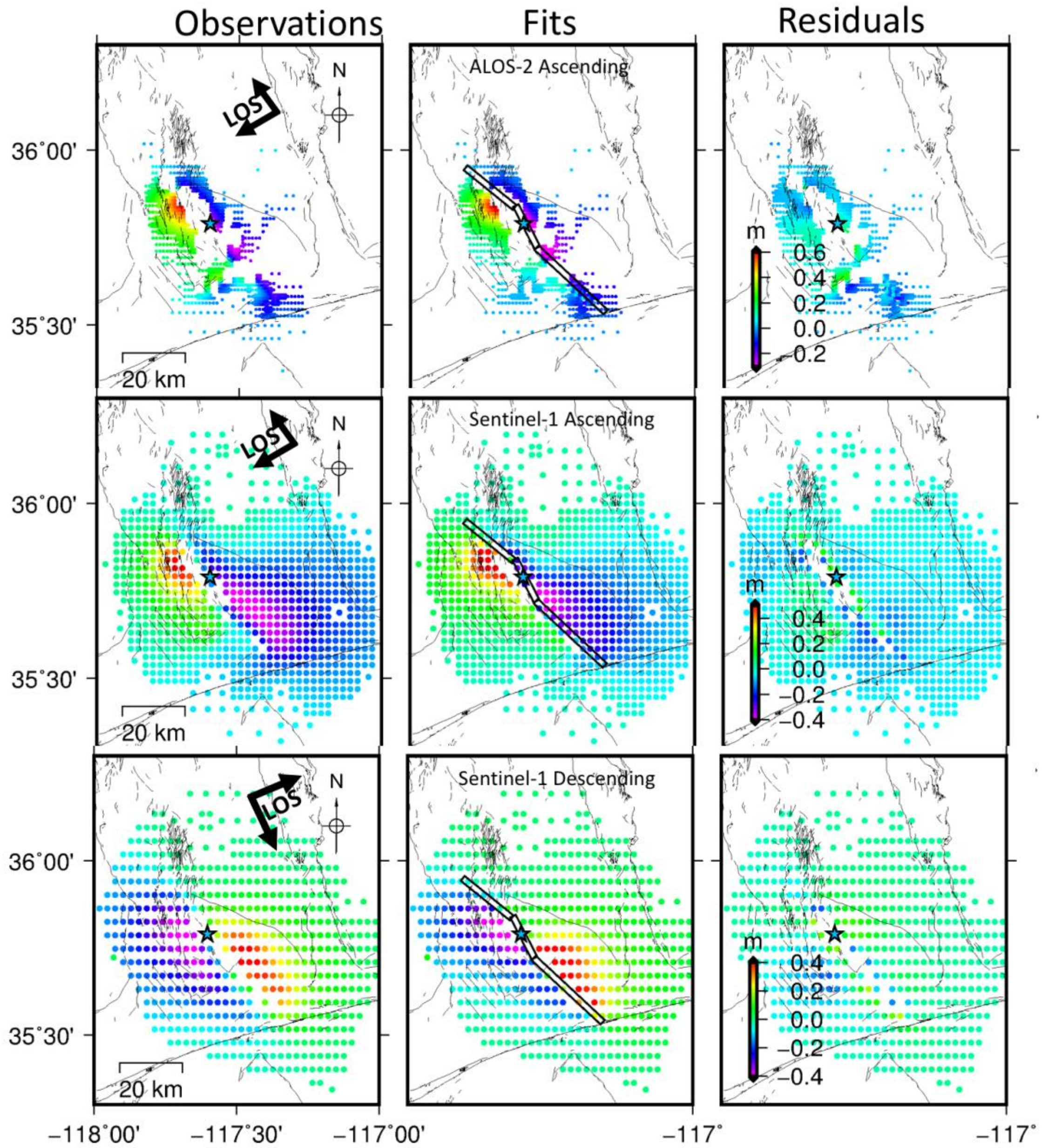

Supplementary Figure 8: InSAR observations (after decimation), model fits and residuals for the 6 July M_W 7.1 event. The top, middle, and bottom panels are results for ALOS-2 ascending track (from April 16, 2018 to July 8, 2019), Sentinel-1 ascending (from July 4, 2019 to July 10, 2019) and descending (from July 4, 2019 to July 16, 2019) tracks. Black rectangular outlines the surface projections of faults adopted for finite source inversion, blue star locates the epicentre.

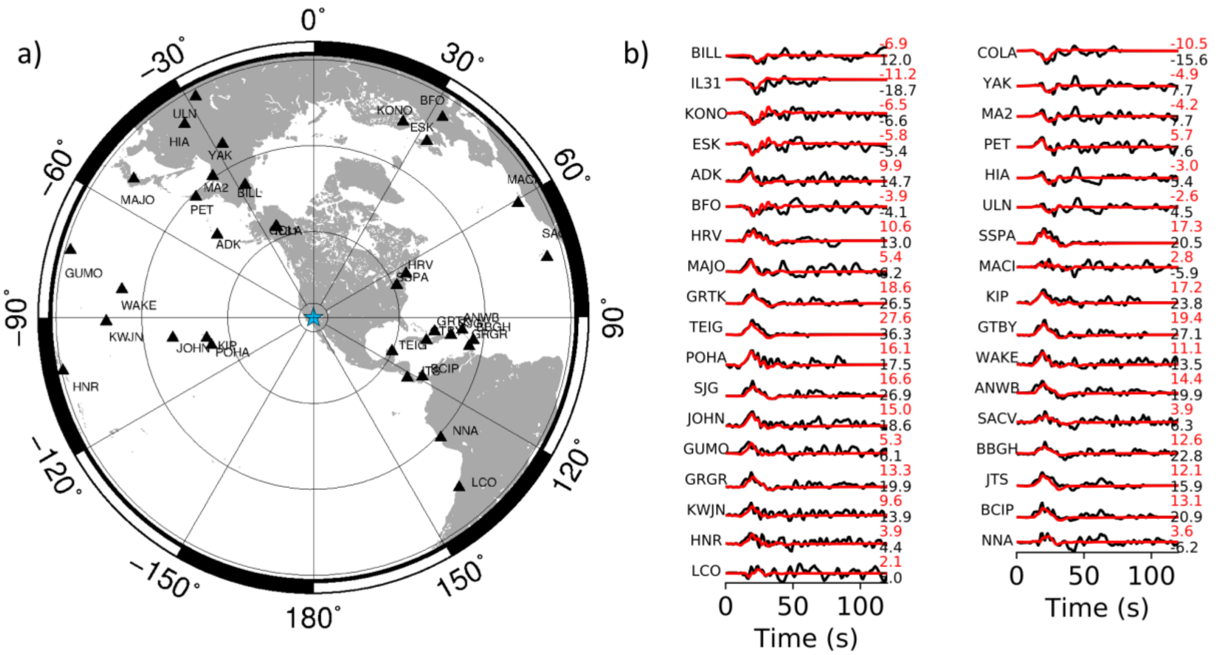

Supplementary Figure 9: Teleseismic station distribution a) and P wave observations and fits b) for the M_w 7.1 event. Stars denote the epicenter, black and red lines are observations and synthetics, respectively. The numbers at right indicate the maximum amplitude values for each waveform with 10^{-6} m as units for teleseismic datasets.

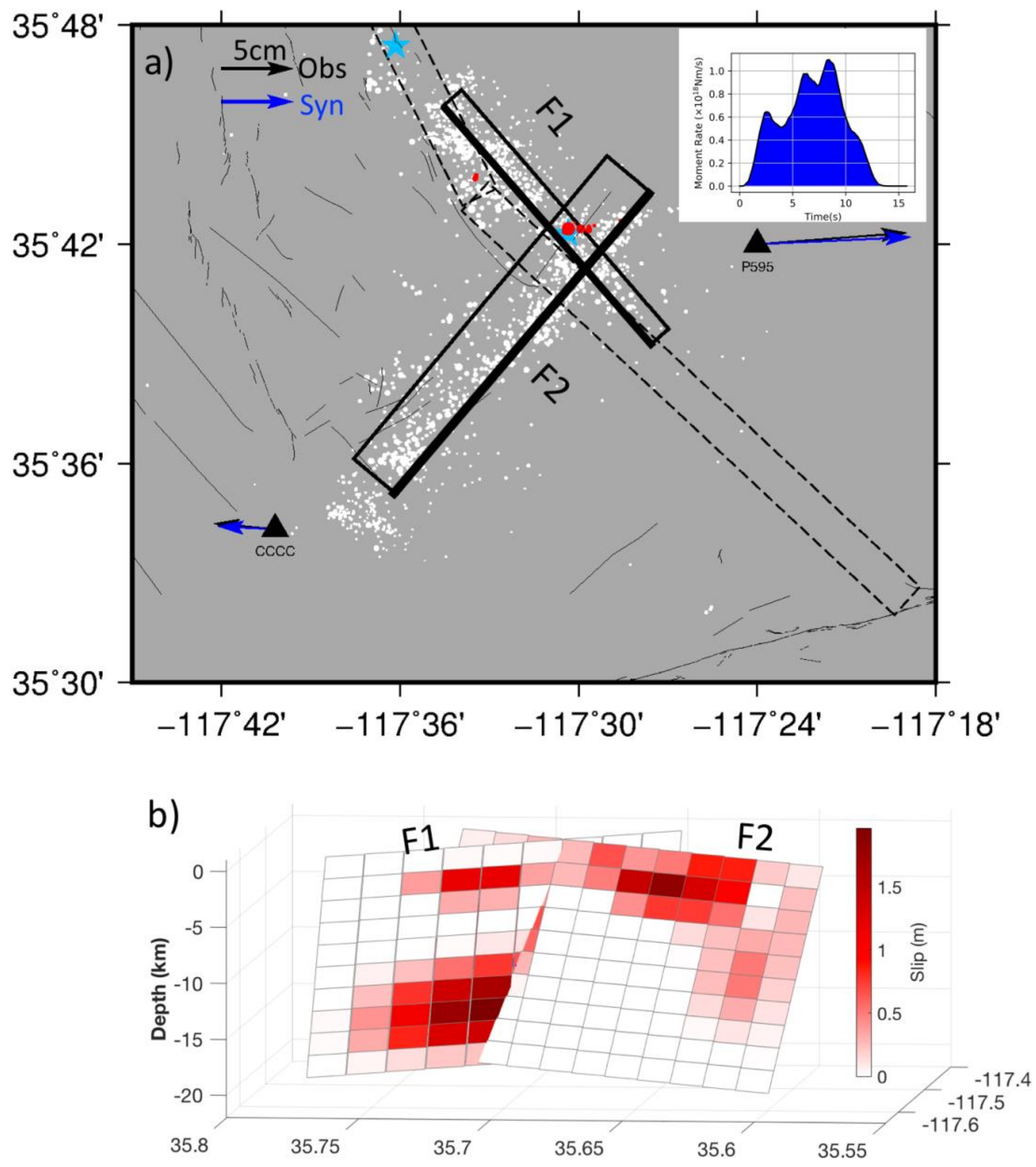

Supplementary Figure 10: Source model of the M_w 6.5 earthquake of July 4, 2019. a) Co-seismic GPS observations (black arrow) and fits (blue arrow). White dots show seismicity after the M_w 6.5 event until the M_w 7.1 event of July 6, red dots are foreshocks. The catalogue is provided by Southern California Earthquake Centre. Solid and dashed rectangle outlines projections of the faults adopted in this study for M_w 6.5 and M_w 7.1 event, respectively, and the thick edges indicate the surface. Right top corner, moment rate function derived. b) Model of the slip distribution for fault F1 and F2, the blue pentagram shows the hypocenter location.

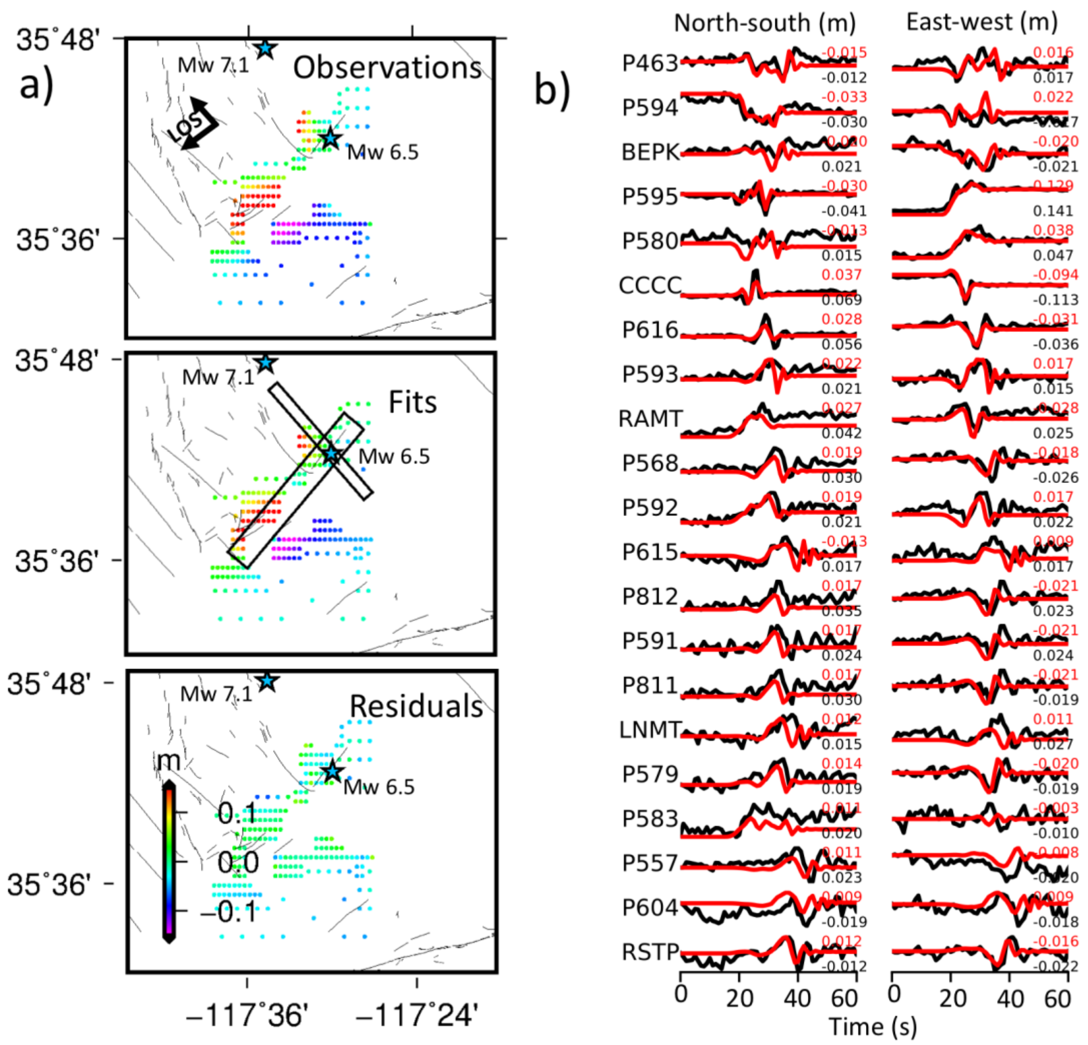

Supplementary Figure 11: InSAR and high-rate GPS data fits of M_w 6.5 earthquake of July 4, 2019. a) For top to bottom: InSAR observations, fits and residuals. Solid rectangle outlines projections of the faults adopted for inversion. Blue stars are epicenters. The observations here are actually residuals of ALOS-2 observations against the Mw 7.1 event. b) High-rate GPS observations (black) and fits (red). The numbers at right indicate the maximum amplitude values for each waveform. See the station distribution in Supplementary Fig. S7a.

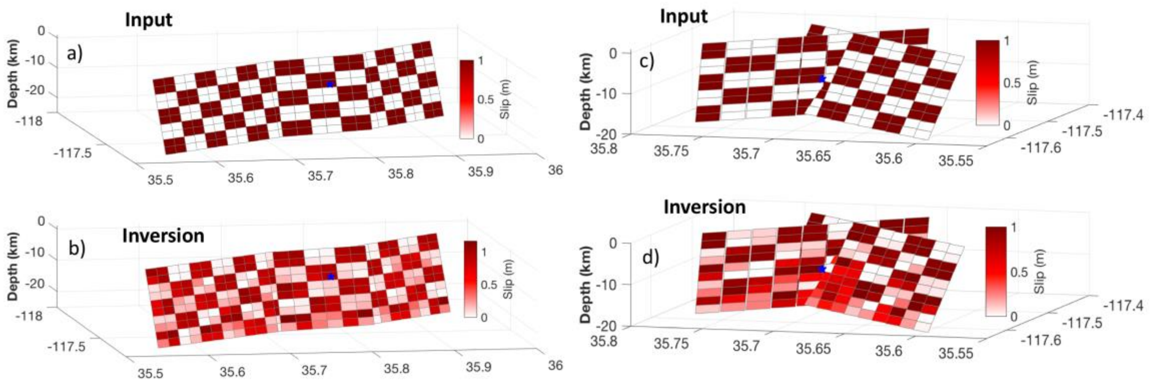

Supplementary Figure 12: **Checkerboard tests for the M_w 7.1 (a), (b) and M_w 6.5 (c), (d) events**. Synthetic static GPS offsets, InSAR measurements, high-rate GPS and P wave displacements are generated by synthetic slip distribution with 1m of slip distributed over every 2×2 patches a), c). Blue star denotes epicenter.

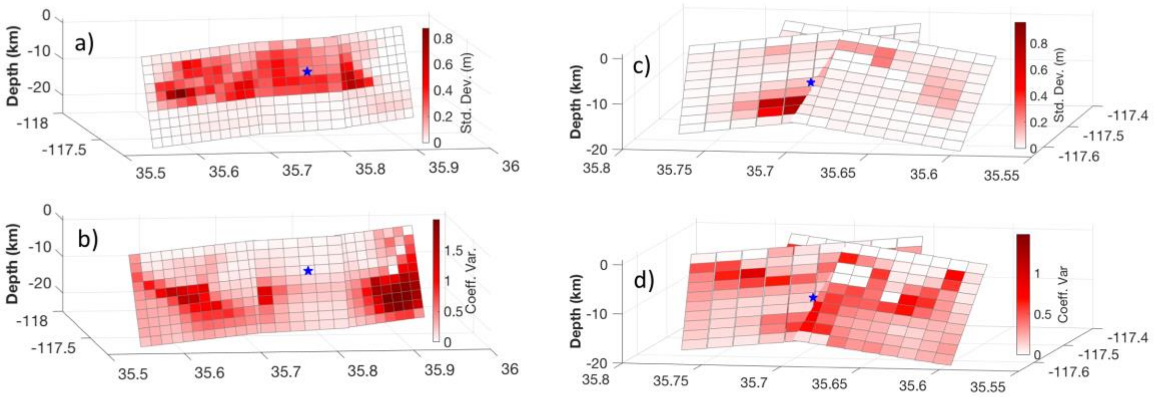

Supplementary Figure 13: **Jackknife testing for the M**w **7.1 (a), (b) and M**w **6.5 (c), (d) events**. Results of removing 20 % of the datasets 100 times. a) and c) are the standard deviation, b) and d) are the coefficient of variation. Blue star denotes epicenter.

Supplementary Tables.

Supplementary Table 1.

Fault geometries for M_w 6.5 and M_w 7.1 event finite source inversion

Event / Parameter	M_w 6.5		M_w 7.1		
	F1	F2	F1	F2	F3
**Strike**	319°	220°	313°	333°	309°
**Dip**	86°	81°	85°		
**Rake**	[155° 205°]	[-25° 25°]	[155° 205°]		
**Patch Size**	2.0×2.0 km^2		2.5×2.5 km^2		
**Dimension**	18×20 km^2	22×20 km^2	30×25 km^2	15×25 km^2	20×10 km^2

Supplementary Table 2.

1-D velocity model for Green's function computation

Thickness (km)	P-velocity (km/s)	S-velocity (km/s)	Density (g/cm^3)	QP	QS
2.50	4.50	2.60	2.10	1000	500
3.00	6.05	3.50	2.75	1000	500
10.50	6.23	3.60	2.80	1000	500
19.00	6.75	3.90	3.05	1000	500
