# Supplementary material for: Cascading and pulse-like ruptures during the 2019 Ridgecrest earthquakes in the Eastern California Shear Zone
Source: Nat Commun. 2020 Jan 7;11:22. doi: 10.1038/s41467-019-13750-w (PMC6946662; doi:10.1038/s41467-019-13750-w)
Supplement: Supplementary file 3 — Description of Additional Supplementary Files [file 41467_2019_13750_MOESM3_ESM.pdf]

Description of Additional Supplementary Files

Supplementary Movie 1

Description: Animation “Slip_Rate_Movie” showing the propagation of the slip-rate at every one second derived from the joint inversion of the teleseismic P waveforms, high-rate GPS, static GPS and InSAR data. The blue star is the hypocenter, and the red dots show the patch centers of surface ruptures. The cumulative slip distribution from the model is shown in Figure 3b.
