# Supplementary material for: Cascading and pulse-like ruptures during the 2019 Ridgecrest earthquakes in the Eastern California Shear Zone
Source: Nat Commun. 2020 Jan 7;11:22. doi: 10.1038/s41467-019-13750-w (PMC6946662; doi:10.1038/s41467-019-13750-w)
Supplement: Supplementary file 2 — Peer Review [file 41467_2019_13750_MOESM2_ESM.pdf]

Reviewers' comments:

Reviewer #1 (Remarks to the Author):

This paper combines optical correlation, GPS, and InSAR data to construct kinematic slip models for the July 4 M6.5 and July 5 M7.1 earthquake sequence. The results look generally excellent and provide a very nice time history of the unfolding sequence of events on the conjugate pair of faults involved, which provide another great example of cross-fault triggering. Overall, the data and interpretations look sound, except as discussed below regarding source dimensions, and these results should be published after the numerous minor issues are corrected, as detailed in the attached Word version. I believe it will be of interest to a broad audience.

The Manuscript required a substantial amount of editing / word-smithing so I took the liberty to correct the numerous minor syntax errors, poor word choices and misspellings in the Word version (tracked) because I think the data are of generally of excellent quality and it will help the authors expedite revisions.

There are several errors in the introduction that should be corrected. I have commented on these in the Word version. They don't in any way affect the results and conclusions presented in this paper, but they should be addressed so that the entire paper is factually correct.

I do see some minor inconsistencies in the relationships between average displacement, fault length, and estimation of moment release. For the Mw6.5 event, the authors estimate a 15 km-long rupture (line 123) with about 55 cm of average slip (line 124). From this, I calculate a moment of about 2.8E18 Nm (assuming 12 km of seismogenic width), which calculates to a Mw6.3. I think they may have underestimate slip at depth or rupture length. Their moment estimate of 7.43E18 Nm, along with a 15 km rupture length with 55 cm average slip, would require a rupture depth of over 30 km, which is not realistic. Similarly, they attribute the M7.1 with 7.43E19 Nm, which is consistent with Mw7.1. However, assuming 12 km of seismogenic thickness and applying their 60 km rupture length with 1.6 m average slip yields about half of the moment. I haven't looked at the D90 seismicity depth but I doubt this rupture extended to 24 km depth, so I suspect the average displacement was higher.

Best regards to the authors, Tom Rockwell

Reviewer #2 (Remarks to the Author):

Chen et al. present a forensic geodetic/seismological study of the recent major earthquakes that occurred near Ridgecrest, California. Overall, the study is pretty solid, with a wide variety of different observations used to constrain the two largest earthquakes, an initial Mw 6.5 event followed by a Mw 7.1 event less than two days later. The paper is also generally well illustrated. There are some omissions on the technical side, which I detail below, but whilst these are essential for readers to assess the uncertainties inherent in the modelling results and should be included, they are unlikely to alter the overall robustness of the key results. The paper also has numerous typographical and grammatical errors throughout, which need to be fixed- I don't list them all below, and they do affect comprehension of the paper, but it good to with one more thorough proof-read by all authors.

However, even if these issues were addressed, I feel this paper is probably not novel enough or of wide enough interest to warrant publication in Nature Communication. This kind of forensic earthquake investigation is now pretty routine for any major, shallow, onshore event, and therefore these papers live or die on their discussion sections and the implications for understanding earthquakes and tectonic processes in general. This paper presents a very good, seismo-geodetic study of the Ridgecrest earthquakes, but is mainly of broader interest simply because these are recent

events in the Western US. There is no major novel observations or improved understanding of earthquakes. There are three themes to the framing, discussion and conclusions which are:

1) the tectonic context of the earthquakes in an area of high geodetic strain-rate but low geological slip rates;
2) the observation of pulse-like rupture in the kinematic slip model;
3) the termination of the rupture at the intersection with another fault at one end, and a volcanic area at the other end.

However, the implications of the Ridgecrest earthquakes for these three topics are not explored in detail, and any such discussion is very qualitative and speculative. There are no concrete conclusions of especial interest relating to these issues. The observation of pulse-like rupture is probably the most interesting of the three, but the authors themselves acknowledge there have already been multiple observations of this process from geodetic data, and the discussion of what these earthquakes might imply for the underlying physical rupture process is brief, speculative, and not very convincing. To remedy this, the paper would probably need a major addition in terms of exploration of the occurrence of pulse-like rupture using a physics-based rupture model (rather than simply describing it using a kinematic model).

So therefore, on this basis, I cannot recommend this paper for publication in Nature Communications. However, this is by no means a judgement of the merit of the scientific content or the obvious large amount of work that has gone into this study- I understand how much effort goes into producing a study like this. With the few technical issues/omissions sorted out (which I detail below), this will be an excellent and valuable forensic study of these earthquakes, but is more suitable for another journal in terms of the results and implications. I look forward to seeing it in published form.

The technical issues/omissions are as follows:

1) None of the modelling includes any discussion of uncertainties. An inverse solution is only useful if it has uncertainties associated with it. These uncertainties should be mentioned in the main text where appropriate (for example when quoting the M0 of the events), and should be presented in detail in additional figures in the supplementary material. This is entirely standard for any earthquake study like this.

2) It is not entirely clear how the fault geometry has been constrained. A W-phase moment tensor is mentioned- is this the authors' own solution or one retrieved from a catalog? This needs clarification and either reference to a supplementary figure or reference to the source of the moment tensor.

3) There are no details of the InSAR processing, and I had to look in the supplementary material to find the dates spanned by the InSAR data. I recommend that data coverage, both in space and time is shown visually on Figure 1 for SAR and optical datasets. I would also like to see the full wrapped interferograms as well as the subsampled data in the supplementary material. This is often the only way to assess whether due care has been taken in terms of identifying/fixing unwrapping errors etc., which can have a huge impact on modelling.

4) There is no exploration or discussion of how the choice of Laplacian smoothing factor affects the results. I'd like to see a visual exploration of this in supplementary material.

5) Likewise, although it's stated that an equal weighting of the different datasets fits them all well, I'd like to see trade-off curves of weighted RMS misfit for the different datasets as these relative weightings are changed, justifying this choice.

6) The modelling approach of inverting for the Mw7.1 first, and then for the Mw6.5 from the residuals

almost certainly exerts a significant bias on both solutions, especially the latter. A joint inversion would be much better, as some datasets constrain both earthquakes, and some constrain just one.

Reviewer #3 (Remarks to the Author):

Summary: This is quick summary of the Ridgecrest earthquakes based on synthesis of remotely sensed data from GPS, InSAR, optical imagery, and seismology. The value of this paper is to confirm that the two ruptures on July 4 and 5 occurred on distinct planes, recovery of a slip function for both of these, and recovery of time-source function for the larger of the two ruptures. This function shows an interesting transition from crack-like to pulse-like rupture that may correlate with pre-stress condition. This event is also part of ongoing cluster in the ECSZ, the drivers of which are not yet settled.

This is a valuable contribution for the synthesis of data and a first look at this pair of events. I would like to see more concrete analysis of the stress conditions on the M 7.1 rupture, such as a comparison of fault strike with regional stress direction.

Here are some detailed comments keyed to sections of the paper:

Abstract: It is not entirely correct to refer to these faults as previously unrecognized. The presence of active dextral and sinistral faults in this area was recognized, as well as the importance of the Airport Lake fault, which ruptured in the M7.1 event. But the extent and connectivity of faults was poorly known.

Line 77: Missing word: pixel resolution (?)

Line 152: Please use MKS units throughout (MPa, not bars)

Line 173: Please explain more concretely how might the strike of F1 and F3 lead to lower initial stress? Is this an argument based on the azimuth of stress here? I think the discussion of fault strike and stress should start at line 179. As currently written, the discussion starts with fault strike, moves immediately to Coulomb stress change, and then returns to fault strike as a cause.

Line 193: of

Line 196: Provide a more concrete explanation of how this event will be used to test dynamic rupture models, e.g. the transition from crack- to pulse-like rupture, and the constraint on pre-stress conditions from rupture geometry.

Figure 1. An overview map of major historical surface ruptures should also include the San Jacinto fault.

Figure 3. The Cost Volcanic Field lies more to the NW than shown. The Quaternary volcanic centers lie closer to the isolated cloud of seismicity.

Reviewers' comments:

Reviewer #1 (Remarks to the Author):

This paper combines optical correlation, GPS, andInSAR data to construct kinematic slip models for the July 4 M6.5 and July 5 M7.1 earthquake sequence. The results look generally excellent and provide a very nice time history of the unfolding sequence of events on the conjugate pair of faults involved, which provide another great example of cross-fault triggering. Overall, the data and interpretations look sound, except as discussed below regarding source dimensions, and these results should be published after the numerous minor issues are corrected, as detailed in the attached Word version. I believe it will be of interest to a broad audience.

The Manuscript required a substantial amount of editing / word-smithing so I took the liberty to correct the numerous minor syntax errors, poor word choices and misspellings in the Word version (tracked) because I think the data are of generally of excellent quality and it will help the authors expedite revisions.

Response: Thanks for your positive comments and careful reading. We apologize for the syntax errors and misspellings. We have made all the edits suggested by the reviewer and checked the editing of the manuscript carefully. Regarding the source dimension, we have now clarified that most of the rupture is blind and that the 15km length refers only to the surface rupture trace, which is only a fraction of the total length of the rupture.

1. There is also the M6.5 Manix Lake earthquake in 1947, as well as a couple ~M6 events in 1975 and 1979 – these are all part of the cluster

Response: We had chosen to report only M>7 events to avoid cluttering the figure. This is specified in the figure caption. We now mention the M6.5 Manix Lake earthquake and the M6 events of 1975 and 1979 in the text.

2. Much of the slip actually joins the southernmost San Andreas fault in the Salton Trough and is transferred to the Imperial fault, not at the tip of the Gulf.

Response: We have now revised our statements as: “and joins with it in the Salton Trough”

3. Most…yes. But only if you include the San Jacinto fault as part of the San Andreas fault? West of the SJF, the Elsinore and coastal system of faults accommodate as much as 20% of the plate motion. Also, where does 46 mm/yr come from? New citation? 49 mm/yr is more generally accepted.

Response: The 46 mm/yr has been corrected to 49 mm/yr both in the manuscript and Fig. 1.

4. This is incorrect unless you include all faults west of the SAF. The point is that the ECSZ has been very active historically, as well as throughout the latest Holocene, and exhibits a higher rate than the long-term geologic rate. Be accurate on where the rest of the plate Boundary shear is concentrated.

Response: To be consistent with the previous paragraph we include the San Jacinto fault and exclude the borderland faults to the west. The fraction of the relative plate motion taken up by the SAF system is then over 60%. Se we now write ‘compared to more than 60% on the SAF system’

5. This RI would only apply to the Garlock fault, which sustains a long-term slip rate of 5-7 mm/yr. Every fault in the ECSZ that has been studied for its paleoseismology has exhibited a RI of 6-10 ka or longer (Rockwell et al., 1990 and other refs).

Response: We are now more accurate and use the proposed wording. We refer to Rockwell et al., 1990 in addition to the other refs, as this is appropriate.

6. It's a double couple. The focal mechanism can't distinguish RL from LL. It would only be the earliest aftershocks.

Response: We have removed the "the first motion focal mechanism" as we indeed show only the moment tensor and can make our point simply based on the epicentral location of the M6.5 event and of its earliest aftershocks.

7. Why? Because strain isn't accumulating because of the high crustal temperatures? Shallow locked zone? Creep?

Response: We have clarified. The sentence now reads: 'High pore pressure due to the hydrothermal activity and higher crustal temperatures could have favored fault creep at shallow depth. The resulting reduced stress level could then have inhibited the propagation of the rupture during the M_w7.1 event. It could have also inhibited aftershocks, as was also observed at Aso volcano following the Kumamoto earthquake, as well as remotely triggered earthquakes'.

8. Was not the Garlock "unclamped" by the M7.1?

Response: This is correct. We added: 'The Garlock fault itself was unclamped locally, mostly west of its intersection with the M_w7.1 rupture.'

9. Need to present the list of citations in a uniform fashion

Response: Done.

10. There are many more surface ruptures on this system of faults since 1850. Qualify with M>7 or include 1918 (M6.9), 1940 (M7), 1947 (M6.5), 1956 (M6.5), 1968 (M6.5), 1971 (M6.7), 1979 (M6.7), 1987 (M6.6), and others. Include 1892 (M7.2) on the Laguna Salada fault (Rockwell et al., 2015)
.

Response: We have limited to the surface ruptures caused by M>7 earthquakes and included the 1892 M7.2 event in Fig. 1, add Rockwell et al., 2015 to the reference list.

11. I do see some minor inconsistencies in the relationships between average displacement, fault length, and estimation of moment release. For the Mw6.5 event, the authors estimate a 15 km-long rupture (line 123) with about 55 cm of average slip (line 124). From this, I calculate a moment of about 2.8E18 Nm (assuming 12 km of seismogenic width), which calculates to a Mw6.3. I think they may have underestimate slip at depth or rupture length. Their moment estimate of 7.43E18 Nm, along with a 15 km rupture length with 55 cm average slip, would require a rupture depth of over 30 km, which is not realistic. Similarly, they attribute the M7.1 with 7.43E19 Nm, which is consistent with Mw7.1. However, assuming 12 km of seismogenic thickness and applying their 60 km rupture length

with 1.6 m average slip yields about half of the moment. I haven't looked at the D90 seismicity depth but I doubt this rupture extended to 24 km depth, so I suspect the average displacement was higher.

Response: The 15 km-long rupture with about 55 cm of average slip is only the surface rupture that revealed by the optical images, not the whole slip (of which mostly buried) jointly inverted from geodetic and seismic data. There is thus no inconsistency. We have clarified this point to avoid a possible misunderstanding.

Reviewer #2 (Remarks to the Author):

Chen et al. present a forensic geodetic/seismological study of the recent major earthquakes that occurred near Ridgecrest, California. Overall, the study is pretty solid, with a wide variety of different observations used to constrain the two largest earthquakes, an initial Mw 6.5 event followed by a Mw 7.1 event less than two days later. The paper is also generally well-illustrated. There are some omissions on the technical side, which I detail below, but whilst these are essential for readers to assess the uncertainties inherent in the modelling results and should be included, they are unlikely to alter the overall robustness of the key results. The paper also has numerous typographical and grammatical errors throughout, which need to be fixed- I don't list them all below, and they do affect comprehension of the paper, but it good to with one more thorough proof-read by all authors.

However, even if these issues were addressed, I feel this paper is probably not novel enough or of wide enough interest to warrant publication in Nature Communication. This kind of forensic earthquake investigation is now pretty routine for any major, shallow, onshore event, and therefore these papers live or die on their discussion sections and the implications for understanding earthquakes and tectonic processes in general. This paper presents a very good, seismo-geodetic study of the Ridgecrest earthquakes, but is mainly of broader interest simply because these are recent events in the Western US. There is no major novel observations or improved understanding of earthquakes. There are three themes to the framing, discussion and conclusions which are:

1) the tectonic context of the earthquakes in an area of high geodetic strain-rate but low geological slip rates;
2) the observation of pulse-like rupture in the kinematic slip model;
3) the termination of the rupture at the intersection with another fault at one end, and a volcanic area at the other end.

However, the implications of the Ridgecrest earthquakes for these three topics are not explored in detail, and any such discussion is very qualitative and speculative. There are no concrete conclusions of especial interest relating to these issues. The observation of pulse-like rupture is probably the most interesting of the three, but the authors themselves acknowledge there have already been multiple observations of this process from geodetic data, and the discussion of what these earthquakes might imply for the underlying physical rupture process is brief, speculative, and not very convincing. To remedy this, the paper would probably need a major addition in terms of exploration of the occurrence of pulse-like rupture using a physics-based rupture model (rather than simply describing it using a kinematic model).

So therefore, on this basis, I cannot recommend this paper for publication in Nature Communications.

However, this is by no means a judgement of the merit of the scientific content or the obvious large amount of work that has gone into this study- I understand how much effort goes into producing a study like this. With the few technical issues/omissions sorted out (which I detail below), this will be an excellent and valuable forensic study of these earthquakes, but is more suitable for another journal in terms of the results and implications. I look forward to seeing it in published form.

Response: Thank you for your comments. We apologize for the poor editing of our initial submission. We believe most of the typographical and grammatical errors have now been fixed. We address your technical concerns below.

1) None of the modelling includes any discussion of uncertainties. An inverse solution is only useful if it has uncertainties associated with it. These uncertainties should be mentioned in the main text where appropriate (for example when quoting the M0 of the events), and should be presented in detail in additional figures in the supplementary material. This is entirely standard for any earthquake study like this.

Response: To estimate the resolution of the inversion, we conducted checkerboard tests which are included as a supplementary Figure. The Figure is inserted below for the M_w 7.1 (left) and the M_w6.5 (right).

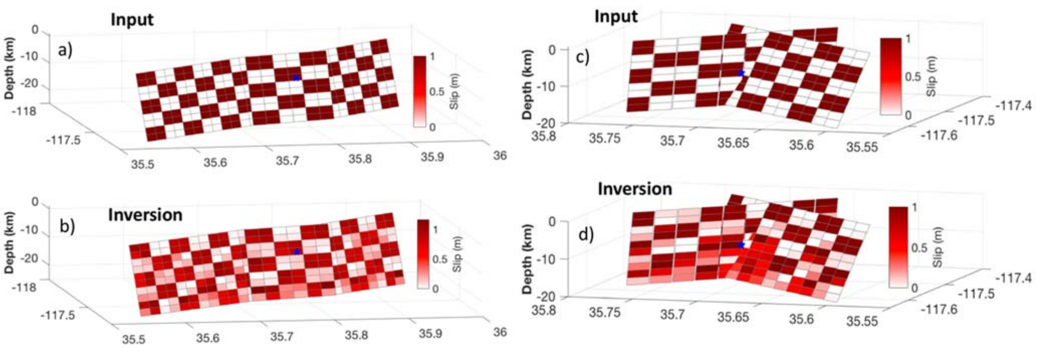

2) It is not entirely clear how the fault geometry has been constrained. A W-phase moment tensor is mentioned- is this the author’s own solution or one retrieved from a catalog? This needs clarification and either reference to a supplementary figure or reference to the source of the moment tensor.

Response: We have clarified that the W-phase moment tensor is from USGS event web page.

3) There are no details of the InSAR processing, and I had to look in the supplementary material to find the dates spanned by the InSAR data. I recommend that data coverage, both in space and time is shown visually on Figure 1 for SAR and optical datasets. I would also like to see the full wrapped interferograms as well as the subsampled data in the supplementary material. This is often the only way to assess whether due care has been taken in terms of identifying/fixing unwrapping errors etc., which can have a huge impact on modelling.

Response: We now show the footprint of the SAR images in Supplementary Figure S6. We used the InSAR data that we were processed and posted online by the JPL ARIA team ((https://aria-share.jpl.nasa.gov/20190704-0705-Searles_Valley_CA_EQs/Interferograms/). Very similar data were produced and posted by UCSD (https://topex.ucsd.edu/SV_7.1/index.html). We have added a brief paragraph in the Method section to present the data and we refer to two publications for details about the processing.

After a personal communication with Cunren Liang, from the ARIA team at JPL/Caltech, the unwrapping errors are believed to be negligible, especially for ALOS-2, which has sparse fringes. The wrapped interferograms are provided by ARIA only for the ALOS-2 data. We show interferograms before and after unwrapping below. As we can see, ARIA already masks out the points that may not be correctly.

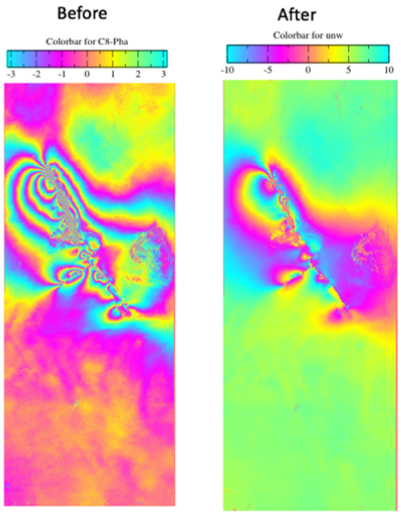

4) There is no exploration or discussion of how the choice of Laplacian smoothing factor affects the results. I'd like to see a visual exploration of this in supplementary material.

Response: For the joint inversion, we ran 25 inversions with different spatial and temporal smoothing and used the Akaike's Bayesian Information Criterion (ABIC, Sekiguchi et al., 2000) to select the preferred model. The figure below, which is included in the supplements, show the ABIC against different smoothing factors is depicted in supplementary figure and the inversions from the weakest and strongest smoothing are also shown. See below.

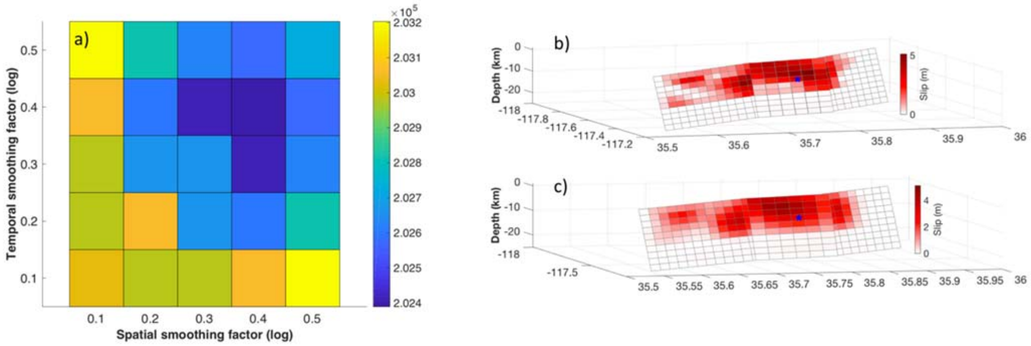

5) Likewise, although it's stated that an equal weighting of the different datasets fits them all well, I'd like to see trade-off curves of weighted RMS misfit for the different datasets as these relative weightings are changed, justifying this choice.

Response: The trade-off curves are now provided, which argues for the reasonable equal weighting strategy, see Supplementary Figure as below.

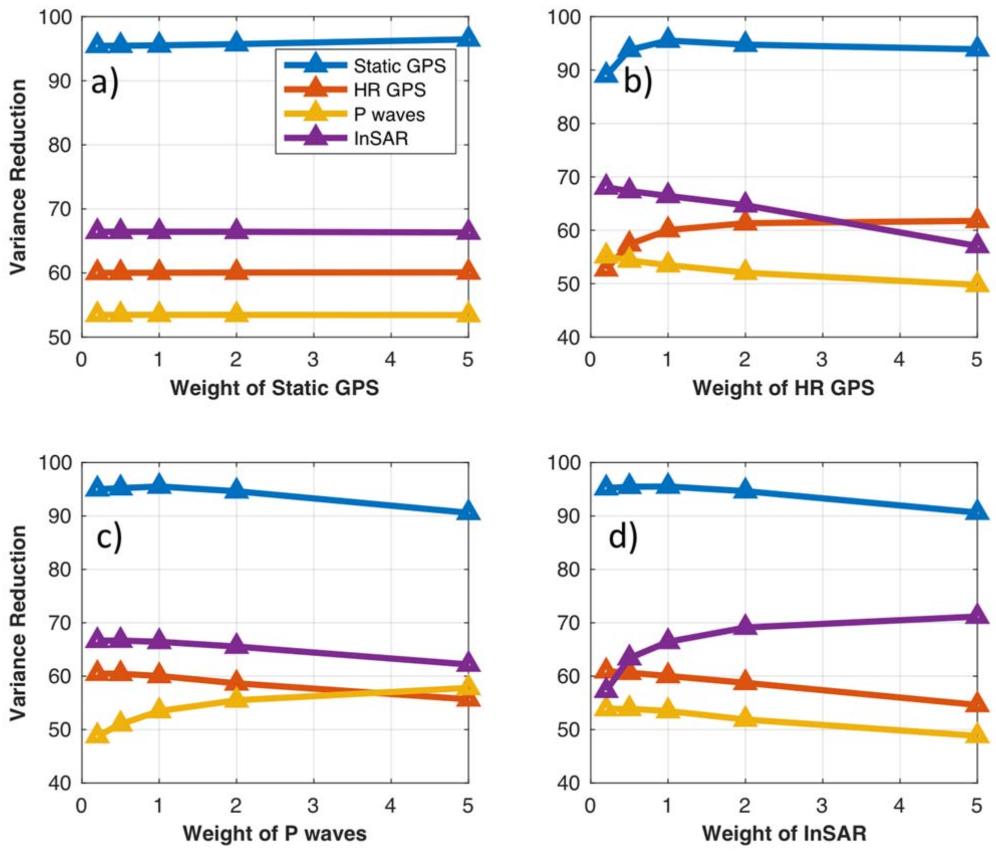

6) The modelling approach of inverting for the Mw7.1 first, and then for the Mw6.5 from the residuals almost certainly exerts a significant bias on both solutions, especially the latter. A joint inversion would be much better, as some datasets constrain both earthquakes, and some constrain just one.

Response: In fact, only the InSAR datasets cannot separate the two events. We however have seismological waveforms, near-field co-seismic high-rate GPS, static GPS measurements and optical image correlation results which all allow separating the two events. Furthermore, considering that the moment of the M_w 7.1 event is almost eight times as large as the moment of the M_w 6.5 event, the bias eventually introduced by the InSAR signal due to the M 6.5 is probably negligible. We don't have a procedure in place to carry out a joint kinematic inversion of the two sources.

Reviewer #3 (Remarks to the Author):

Summary: This is quick summary of the Ridgecrest earthquakes based on synthesis of remotely sensed data from GPS,InSAR, optical imagery, and seismology. The value of this paper is to confirm that the two ruptures on July 4 and 5 occurred on distinct planes, recovery of a slip function for both of these, and recovery of time-source function for the larger of the two ruptures. This function shows an interesting transition from crack-like to pulse-like rupture that may correlate with pre-stress condition. This event is also part of ongoing cluster in the ECSZ, the drivers of which are not yet settled.

This is a valuable contribution for the synthesis of data and a first look at this pair of events. I would like to see more concrete analysis of the stress conditions on the M 7.1 rupture, such as a comparison of fault strike with regional stress direction.

Here are some detailed comments keyed to sections of the paper:

Abstract: It is not entirely correct to refer to these faults as previously unrecognized. The presence of active dextral and sinistral faults in this area was recognized, as well as the importance of the Airport Lake fault, which ruptured in the M7.1 event. But the extent and connectivity of faults was poorly known.
Response: Thanks for the information, we have adjusted what we wrote in that regard and removed the points from the abstract as it is not in any case a key finding.

Line 77: Missing word: pixel resolution (?)
Response: Yes, and we have revised it to
"We selected publicly available Sentinel-2 and Planet Labs with Ground Sample Distance of 10 and 3 meters, respectively"

Line 152: Please use MKS units throughout (MPa, not bars)
Response: Done, we also update the units in Fig. 3c to Mpa.

Line 173: Please explain more concretely how might the strike of F1 and F3 lead to lower initial stress? Is this an argument based on the azimuth of stress here? I think the discussion of fault strike and stress should start at line 179. As currently written, the discussion starts with fault strike, moves immediately to Coulomb stress change, and then returns to fault strike as a cause.
Response: Done

Line 193: of

Response: Done.

Line 196: Provide a more concrete explanation of how this event will be used to test dynamic rupture models, e.g. the transition from crack- to pulse-like rupture, and the constraint on pre-stress conditions from rupture geometry.

Response: We have rephrase and expanded a bit this point. It now reads: 'It reveals a sequence of triggering, where small foreshocks initiated on a secondary fault which eventually produced a complex rupture of two conjugate faults on July 4, leading to the eventually triggering of the M_w 7.1 event on July 5. The larger event evolved from a crack-like to a pulse-like rupture and arrested due to a combination of geometric, and probably hydromechanical effects. These features are noteworthy as they can serve to test and calibrate dynamical simulations of these earthquakes.'

Figure 1. An overview map of major historical surface ruptures should also include the San Jacinto fault.
Response: We opted for showing only ruptures from M>7 events as the information about ruptures from smaller historical events is probably incomplete. We have now clarified that in the caption of Fig. 1.

Figure 3. The Cost Volcanic Field lies more to the NW than shown. The Quaternary volcanic centers lie closer to the isolated cloud of seismicity.

Response: Thank you for noticing the issue. This is corrected.

Reviewers' comments:

Reviewer #1 (Remarks to the Author):

2nd Review of “Cascading and pulse-like ruptures during the 2019 Ridgecrest earthquakes in the East California Shear Zone” by Chen et al.

Dear Editor and Authors.

The second version of this paper is greatly improved but still has a couple things that need to be fixed. Most of the syntax errors have been corrected so the paper is fairly clean in terms of readability. There is still the issue of their description of the rupture in terms of length and average slip vs the calculated moments – I do not think this is completely clear and I have queried this in the “tracked” text (along with the many small suggested edits).

One significant error that must be corrected relates to figure 1 – they have added the 1892 rupture but have located it along the southern San Andreas fault, which is wrong. The rupture occurred along the Laguna Salada fault, which is adjacent to the 2010 rupture in Baja California.

There are also other M7+ earthquakes that have occurred in the historical period that fall within the confines of figure 1. The 1927 M7.2 Point Arguello earthquake was an offshore thrust event. The M7.3 November 22, 1800 earthquake ruptured the south-central San Jacinto fault (Clark strand) (Salisbury et al, 2012). The December 22, 1812 (M7+) earthquake ruptured at least part of the San Andreas fault and most likely part of the northern San Jacinto fault (various citations including Jacoby and Sieh, 1981, Julian Lozos papers, Onderdonk et al., 2017, Rockwell et al., 2015). The earlier Dec 11, 1812 earthquake is purported to also have ruptured the San Andreas fault farther NW (Toppazada ref) and was also M7+. It seems that if they are to exclude earthquakes <M7, then they should at least include all of the M7+ events within the area of figure 1. I am willing to help them locate all of the correct citations (and locations) when I return to the US (and have access to my office computer, as I am currently doing field work in Germany).

Once these details are corrected, I think the manuscript will be ready to go and of broad interest.

Regards, Tom

PS - I am back in the US after October 20 and can assist the authors with any cited details then.

Reviewer #2 (Remarks to the Author):

This is a re-review of Chen et al.’s seismotectonic study of the recent Ridgecrest earthquakes.

My main criticism of the paper has not really been addressed, and I was slightly disappointed that this was pretty much ignored in the rebuttal letter from the authors. To re-iterate: this is a solid forensic study of the Ridgecrest earthquakes (barring the technical omissions, which have now largely been

addressed), but there is no real attempt in the paper to elevate it from a regional earthquake case study, and it is disappointing that there is no real take-home message for understanding earthquake or tectonic processes in general. This is highlighted in particular by the final line of the abstract: “This study provides insight into the factors that controls the mode and extent of seismic ruptures.” which is neither specific nor quantitative, and is a pretty generic and uninformative take-home message. However, I do understand that this is an editorial decision, and so while my recommendation still remains that this paper is not suitable for Nature Communications on this front, I will review the rest of the paper regardless.

I thank the authors for adding new information and supplementary material to address my concerns over the lack of important supporting technical information, and these additions certainly strengthen the results. However, the authors have mixed up model resolution and uncertainty and therefore have not addressed my previous point 1):

"1) None of the modelling includes any discussion of uncertainties. An inverse solution is only useful if it has uncertainties associated with it. These uncertainties should be mentioned in the main text where appropriate (for example when quoting the M0 of the events), and should be presented in detail in additional figures in the supplementary material. This is entirely standard for any earthquake study like this."

The authors added checkerboard tests, which although important, are a way of illustrating the resolution associated with the inverse solution, not the model uncertainties. Just to clarify- the *model resolution* shows how, even with perfect, noise-free data, any ‘true’ model will be ‘blurred’ by the inversion process due to smoothing and under-determined parts of the model space etc., whereas the *model uncertainties* (e.g. the model variance covariance matrix) show how noise in the data is mapped through the inverse problem onto the model. Calculation and some visualisation of model covariance is pretty fundamental to the critical assessment of any inverse model results, and as I stated in my previous review, uncertainties on slip for each patch/time window and for the M0 of the events are important. So unfortunately, this issue has not been addressed and I still recommend that this is remedied prior to publication.

All other issues were addressed, but I also list some grammatical/typographic corrections below:

L15: “struck Searles Valley”

L16: shock -> aftershock

L19: “the Mw 7.1 event. This larger earthquake started as....”. Grammatically it is odd to just refer to “a Mw 7.1” without a noun at the end. An earthquake should always be referred to in the form “a Mw 7.1 earthquake” or “event” etc. This should be corrected throughout, e.g. also at L27 and elsewhere in the main text.

L22: rather -> relatively

L26: “rattled Searles Valley near Ridgecrest...”

L33: “they” is ambiguous and could refer here to the GPS records -> replace with “the earthquakes”.

L36: with -> and

L38: reference for the ARIA project.

L457: Salton Trough

L50: ECSZ,

L56: “associated with” or “assigned to”

L60: delete “though”, and

L70: “associated with”

L75: “publically-available Sentnel-2 and Planet Labs optical satellite images with”

L78: cubesat typo. “managed to acquire” -> “acquired”

L88: “Both datasets clearly show”

L120, 148: cumulated -> total

L124: delete “actually”

L140: The major one before -> The largest earthquake preceding

L151: Mpa -> MPa

L169: “strong velocity-weakening friction on the fault”.

L184: “close to the”

L195: eventually -> eventual. But also repetition with eventually in preceding sentence- suggest rewording these two sentences for clarity.

L196: “possibly arrested” – no strong evidence presented here other than correlation, so this has to come with strong caveats.

L206-208: Repetition in this sentence, probably a copying error here- reword for clarity.

L313: “of high-frequency noise”

L360: “We obtained three focused radar images”. Unclear- is this three SAR images? Or three interferograms? If the latter, better to state here the number of SAR images, not interferograms.

L400-402: weird grammar in this sentence, please reword.

L405: reference for GCMT

Reviewer #3 (Remarks to the Author):

I am content overall with the revisions to the manuscript. The unusual characteristics of this sequence and its possible relationship to loading and pre-stress conditions are more clearly highlighted. It is important to appreciate that this is an early result and that more detailed analyses are sure to follow. The integration of multiple geodetic, seismic, and image differencing datasets is impressive.

Below I provide several line-by-line editing comments. Most of these are easy fixes.

23: Do not capitalize cardinal directions. The North and the South fought the civil war.

62. the rest of the SAF system

68. with exception of the Garlock fault

69. I’m not so sure this statement would apply to faults north of the Garlock, such as the Panamint Valley or Death Valley faults. But for those that have been documented, other than the Garlock fault, most have recurrence intervals exceeding 5 kyr. Perhaps instead of ‘every fault in the ECSZ that has’ the authors should state ‘most faults in the ECSZ that have

75. Why is Ground Sample Distance capitalized?

78 cubesat

83, 86 artifacts

84 why is Digital Elevation Model capitalized?

98. Please be consistent about significant digits, e.g. 1.6±0.2 and 0.30±0.15

123 and 124 delete actually

178 both strike NW (provide actual azimuths in parentheses)

179 which strikes NNW (provide azimuth)

182 encompasses an area containing numerous

184. The northern termination of the 7.1 event is reminiscent

Reviewed by Mike Oskin

Reviewers' comments:

Reviewer #1 (Remarks to the Author):

2nd Review of “Cascading and pulse-like ruptures during the 2019 Ridgecrest earthquakes in the East California Shear Zone” by Chen et al.

Dear Editor and Authors.

The second version of this paper is greatly improved but still has a couple things that need to be fixed. Most of the syntax errors have been corrected so the paper is fairly clean in terms of readability. There is still the issue of their description of the rupture in terms of length and average slip vs the calculated moments – I do not think this is completely clear and I have queried this in the “tracked” text (along with the many small suggested edits).

Response: We have modified the text to clarify further how the moment of the M6.5 event relates to the fault length and measured slip. The plot below illustrates this relationship. The surface slip along the NE striking fault (outlined by the blue box) were directly measured using the optical images. Most of the moment is from the buried slip patch. The original statement was "Our measurements indicate that the Mw 6.5 event ruptured a ~15 km long fault striking NE with an average of 0.55+/-0.08 m of left-lateral slip”. We were referring here to the mean “surface slip”. We have modified the end of the sentence “left-lateral **surface** slip”. In addition where we refer to the moment of that event we inserted ‘, which released most of the moment,’ the revised text reads:
‘According to our model (see Fig. S6), the M_w 6.5 earthquake itself is a compound event which initiated on the right-lateral NW striking, **which released most of the moment**, fault and then activated the left-lateral fault. It released a total moment of 7.43×10^{18} N·m (equivalent to M_w 6.5) in about 10s.’

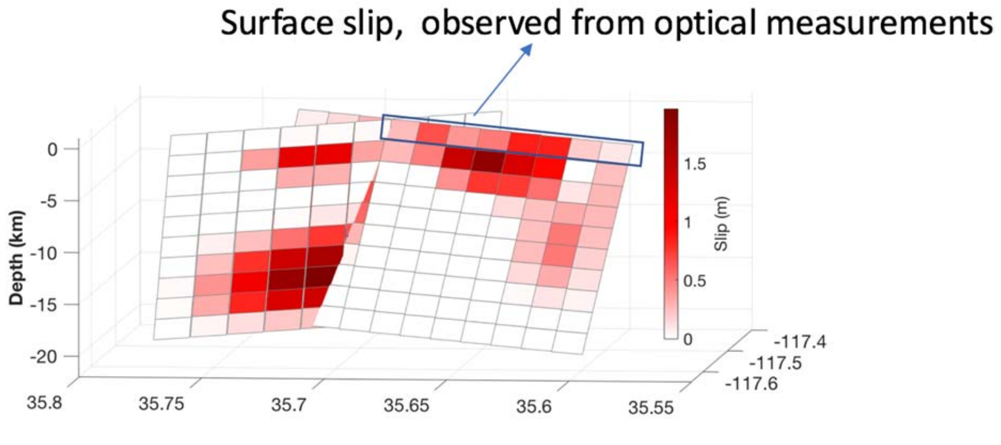

One significant error that must be corrected relates to figure 1 – they have added the 1892 rupture but have located it along the southern San Andreas fault, which is wrong. The rupture occurred along the Laguna Salada fault, which is adjacent to the 2010 rupture in Baja California. There are also other M7+ earthquakes that have occurred in the historical period that fall within the confines of figure 1. The 1927 M7.2 Point Arguello earthquake was an offshore thrust event. The M7.3 November 22, 1800 earthquake ruptured the south-central San Jacinto fault (Clark strand) (Salisbury et al, 2012). The December 22, 1812 (M7+) earthquake ruptured at least part of the San Andreas fault and most likely part of the northern San Jacinto fault (various citations including Jacoby and Sieh, 1981, Julian Lozos papers, Onderdonk et al., 2017, Rockwell et al., 2015). The earlier Dec 11, 1812 earthquake is purported to also have ruptured the San Andreas fault farther NW (Toppazada ref) and was also M7+. It seems that if they are to exclude earthquakes <M7, then they should at least include all of the M7+ events within the area of figure 1. I am willing to help them locate all of the correct citations (and locations) when I return to the US (and have access to my office computer, as I am currently doing field work in Germany).

Response: Thanks for the complete information about the M7+ earthquakes and spotting the error about the 1892 earthquake. We have corrected the error. and added the

estimated ruptures of the 1800 M7.3 earthquake, the 1812 M7.5 earthquake, the 1927 M7.2 earthquake, the 1940 M7.0 Imperial Valley earthquake. We refer to the studies suggested by the reviewer (Lozos, 2016; Toppozada and Branum, 2004; Salisbury et al, 2012)

See revised Figure 1 below:

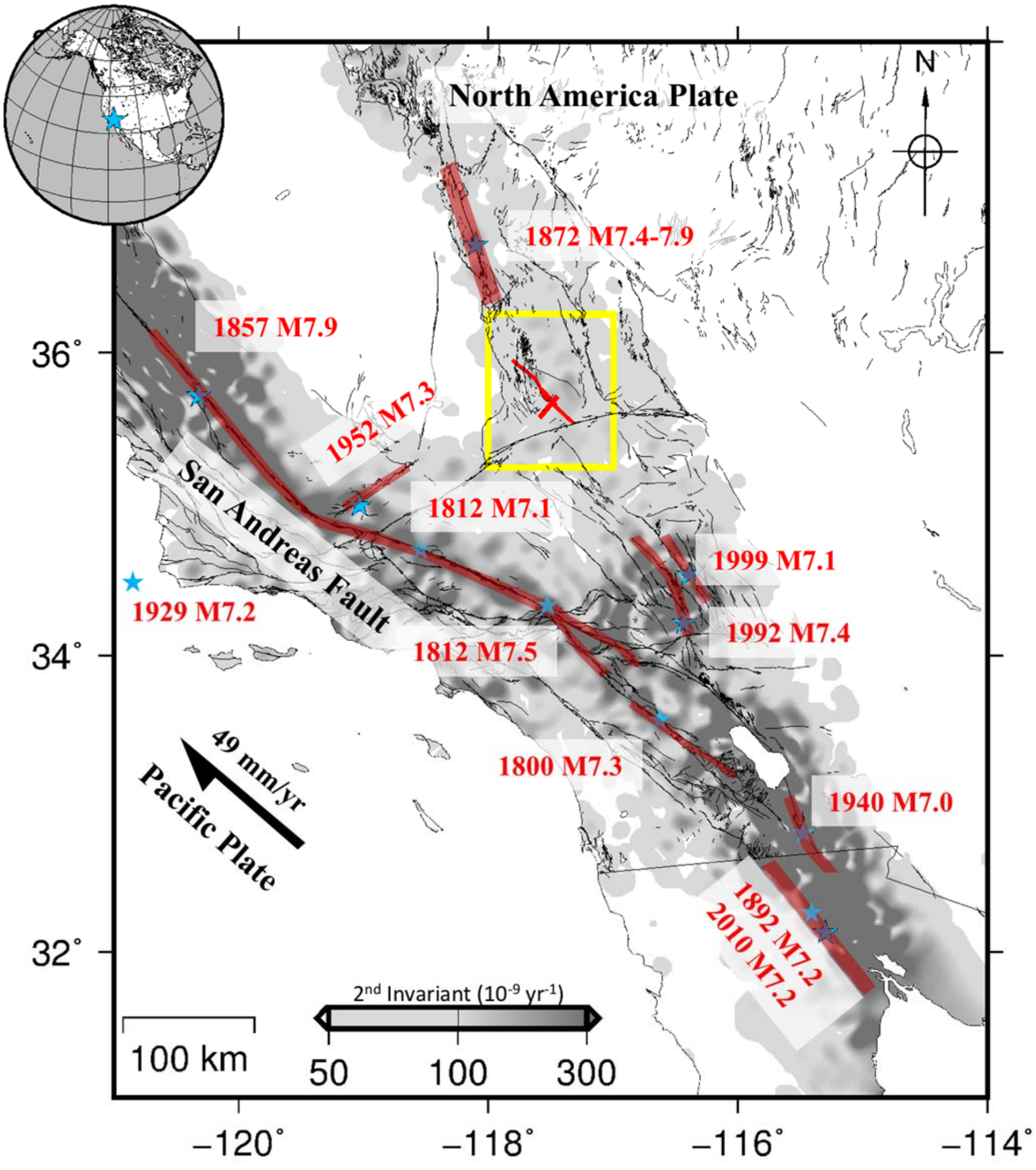

Once these details are corrected, I think the manuscript will be ready to go and of broad interest.

Regards, Tom

Reviewer #2 (Remarks to the Author):

This is a re-review of Chen et al.'s seismotectonic study of the recent Ridgecrest earthquakes.

My main criticism of the paper has not really been addressed, and I was slightly disappointed that this was pretty much ignored in the rebuttal letter from the authors. To re-iterate: this is a solid forensic study of the Ridgecrest earthquakes (barring the technical omissions, which have now largely been addressed), but there is no real attempt in the paper to elevate it from a regional earthquake case study, and it is disappointing that there is no real take-home message for understanding earthquake or tectonic processes in general. This is highlighted in particular by the final line of the abstract: "This study provides insight into the factors that controls the mode and extent of seismic ruptures." which is neither specific nor quantitative, and is a pretty generic and uninformative take-home message.
However, I do understand that this is an editorial decision, and so while my recommendation still remains that this paper is not suitable for Nature Communications on this front, I will review the rest of the paper regardless.

Response: We now emphasize better the take home massages and the broader implications. This was achieved with some re-organization of the manuscript and by adding a few sentences in the conclusion. We are now better emphasizing two points:

- Our details source model show the M7.1 event evolved from evolved from a crack-like to a pulse-like rupture. This evolution correlates with strike changes and the co-seismic stress changes due to the Mw 6.5 event. These features are remarkable (and visible only because of the availability of data of exceptional quality) as they are qualitatively consistent with theory. We are not aware of any prior similar observations.

- The Ridgecrest earthquakes are part of a cluster of historical EQs probably associated to a long-lived strain transient and that the faults just north of Coso or south of the Garlock fault, left unbroken during this cluster, are now closer to failure.

I thank the authors for adding new information and supplementary material to address my concerns over the lack of important supporting technical information, and these additions certainly strengthen the results. However, the authors have mixed up model resolution and uncertainty and therefore have not addressed my previous point 1):

"1) None of the modelling includes any discussion of uncertainties. An inverse solution is only useful if it has uncertainties associated with it. These uncertainties should be mentioned in the main text where appropriate (for example when quoting the M0 of the events), and should be presented in detail in additional figures in the supplementary material. This is entirely standard for any earthquake study like this."

The authors added checkerboard tests, which although important, are a way of illustrating the resolution associated with the inverse solution, not the model uncertainties. Just to clarify- the *model resolution* shows how, even with perfect, noise-free data, any 'true' model will be 'blurred' by the inversion process due to smoothing and under-determined parts of the model space etc., whereas the *model uncertainties* (e.g. the model variance covariance matrix) show how noise in the data is mapped through the inverse problem onto the model. Calculation and some visualisation of model covariance is pretty fundamental to the critical assessment of any inverse model results, and as I stated in my previous review, uncertainties on slip for each patch/time window and for the M0 of the events are important. So unfortunately, this issue has not been addressed and I still recommend that this is remedied prior to publication.

Response: Following your request we now illustrate the uncertainties on the model parameters by showing the results of a Jack-knife test in Fig. S13 which is inserted below:

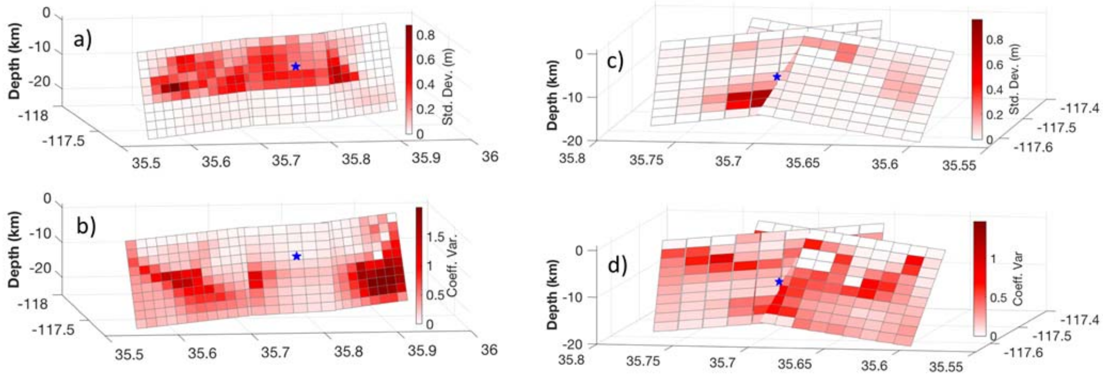

All other issues were addressed, but I also list some grammatical/typographic corrections below:

L15: “struck Searles Valley”
Response: Done

L16: shock -> aftershock
Response: The M_w 7.1 event is considered to be the main shock, not aftershock.

L19: “the Mw 7.1 event. This larger earthquake started as....”. Grammatically it is odd to just refer to “a Mw 7.1” without a noun at the end. An earthquake should always be referred to in the form “a Mw 7.1 earthquake” or “event” etc. This should be corrected throughout, e.g. also at L27 and elsewhere in the main text.
Response: Done.

L22: rather -> relatively
Response: Done

L26: “rattled Searles Valley near Ridgecrest...”
Response: Done.

L33: “they” is ambiguous and could refer here to the GPS records -> replace with “the earthquakes”.
Response: Done.

L36: with -> and
Response: Done.

L38: reference for the ARIA project.
Response: We have added the reference (https://aria.jpl.nasa.gov/).

L457: Salton Trough
Response: Done.

L50: ECSZ,
Response: The comma is now added.

L56: “associated with” or “assigned to”
Response: We have now revised it as “associated with”

L60: delete “though”
Response: Done.

L70: “associated with”
Response: Done

L75: “publically-available Sentnel-2 and Planet Labs optical satellite images with”
Response: Done.

L78: cubesat typo. “managed to acquire” -> “acquired”
Response: The typo has been now fixed.

L88: “Both datasets clearly show”
Response: Done.

L120, 148: cumulated -> total
Response: Done

L124: delete “actually”
Response: Done.

L140: The major one before -> The largest earthquake preceding

Response: Done.

L151: Mpa -> MPa
Response: Done.

L169: “strong velocity-weakening friction on the fault”.
Response: Done.

L184: “close to the”
Response: Done.

L195: eventually -> eventual. But also repetition with eventually in preceding sentence- suggest rewording these two sentences for clarity.
Response: Done.

L196: “possibly arrested” – no strong evidence presented here other than correlation, so this has to come with strong caveats.
Response: Done.

L206-208: Repetition in this sentence, probably a copying error here- reword for clarity.
Response: We have removed the repetition.

L313: “of high-frequency noise”
Response: Done.

L360: “We obtained three focused radar images”. Unclear- is this three SAR images? Or three interferograms? If the latter, better to state here the number of SAR images, not interferograms.
Response: Done.

L400-402: weird grammar in this sentence, please reword.
Response: Done, and now it reads:

For the Mw 6.5 event, in addition to the NE trending rupture that is recovered by the optical images, the distribution of the aftershocks implies that it should have probably ruptured a conjugating fault, and we adopt two fault planes to model this event.

L405: reference for GCMT

Response: Done. We now refer to:

Ekström, G., M. Nettles, and A. M. Dziewonski, The global CMT project 2004-2010: Centroid-moment tensors for 13,017 earthquakes, Phys. Earth Planet. Inter., 200-201, 1-9, 2012. doi:10.1016/j.pepi.2012.04.002

Reviewer #3 (Remarks to the Author):

I am content overall with the revisions to the manuscript. The unusual characteristics of this sequence and its possible relationship to loading and pre-stress conditions are more clearly highlighted. It is important to appreciate that this is an early result and that more detailed analyses are sure to follow. The integration of multiple geodetic, seismic, and image differencing datasets is impressive.

Below I provide several line-by-line editing comments. Most of these are easy fixes.

23: Do not capitalize cardinal directions. The North and the South fought the civil war.
Response: Thank you for your careful proofreading, and it has been fixed.

62. the rest of the SAF system
Response: Done.

68. with exception of the Garlock fault
Response: Done.

69. I’m not so sure this statement would apply to faults north of the Garlock, such as the Panamint Valley or Death Valley faults. But for those that have been documented, other than the Garlock fault, most have recurrence intervals exceeding 5 kyr. Perhaps instead of ‘every fault in the ECSZ that has’ the authors should state ‘most faults in the ECSZ that have
Response: Thank you for the information, and we have modified it to “most faults in the ECSZ that we have”

75. Why is Ground Sample Distance capitalized?
Response: We have now revised it to “ground sample distance”

78 cubesat
Response: Done.

83, 86 artifacts
Response: The plural of artifact is actually “arterfacts”.

84 why is Digital Elevation Model capitalized?
Response: We have now revised it to “digital elevation model”.

98. Please be consistent about significant digits, e.g. 1.6±0.2 and 0.30±0.15
Response: Done.

123 and 124 delete actually
Response: Done.

178 both strike NW (provide actual azimuths in parentheses)
Response: Done.

179 which strikes NNW (provide azimuth)
Response: Done.

182 encompasses an area containing numerous
Response: Done.

184. The northern termination of the 7.1 event is reminiscent
Response: Done.

Reviewed by Mike Oskin

REVIEWERS' COMMENTS:

Reviewer #1 (Remarks to the Author):

I have made several additional minor edits (word choices), which was now easy because the manuscript is clean. This manuscript can be accepted as is, although I'd suggest adding a short paragraph, or at least a couple lines, on the fact that the estimate of Mo based on average surface slip underestimates the total Mo released on the NE striking fault, which implies that there was more slip at depth then observed at the surface. As the authors state, about half of the Mo for this event was apparently on a NW-striking fault that did not rupture to the surface so all of the Mo associated with this sub-event was "blind". This is not a major issue and the authors may wish to ignore this suggestion as it is implied, but not clearly stated.

All other edits are tracked in the attached doc. file.

Finally, Fig. 1 looks great and I should have noticed this earlier, but the 1934 M7.1 Cerro Prieto rupture in Baja California should be added. I will send the authors the amended figure directly, but include here as well (attached)

TKR

Reviewer #2 (Remarks to the Author):

The authors have addressed all of the issues I have raised, and the manuscript is now suitable for publication.

There is one minor error in new text on L464-465, and I suggest rewording as follows:

"...the coefficient of variation (CV) can be a better measure for showing which features are persistent."

REVIEWERS' COMMENTS:

Reviewer #1 (Remarks to the Author):

I have made several additional minor edits (word choices), which was now easy because the manuscript is clean. This manuscript can be accepted as is, although I'd suggest adding a short paragraph, or at least a couple lines, on the fact that the estimate of Mo based on average surface slip underestimates the total Mo released on the NE striking fault, which implies that there was more slip at depth then observed at the surface. As the authors state, about half of the Mo for this event was apparently on a NW-striking fault that did not rupture to the surface so all of the Mo associated with this sub-event was "blind". This is not a major issue and the authors may wish to ignore this suggestion as it is implied, but not clearly stated.

All other edits are tracked in the attached doc. file.

Finally, Fig. 1 looks great and I should have noticed this earlier, but the 1934 M7.1 Cerro Prieto rupture in Baja California should be added. I will send the authors the amended figure directly, but include here as well (attached)

TKR

Response: Thank you for your suggestion. For the moment and surface rupture relationship, we believe that our current version of the text is sufficiently clear to indicate the moment could not be reliably estimated just from the surface measurements.

All the edits are now incorporated in the manuscript.

We have revised Fig. 1 by adding the 1934 M7.1 Cerro Prieto rupture as you suggested, see the follow:

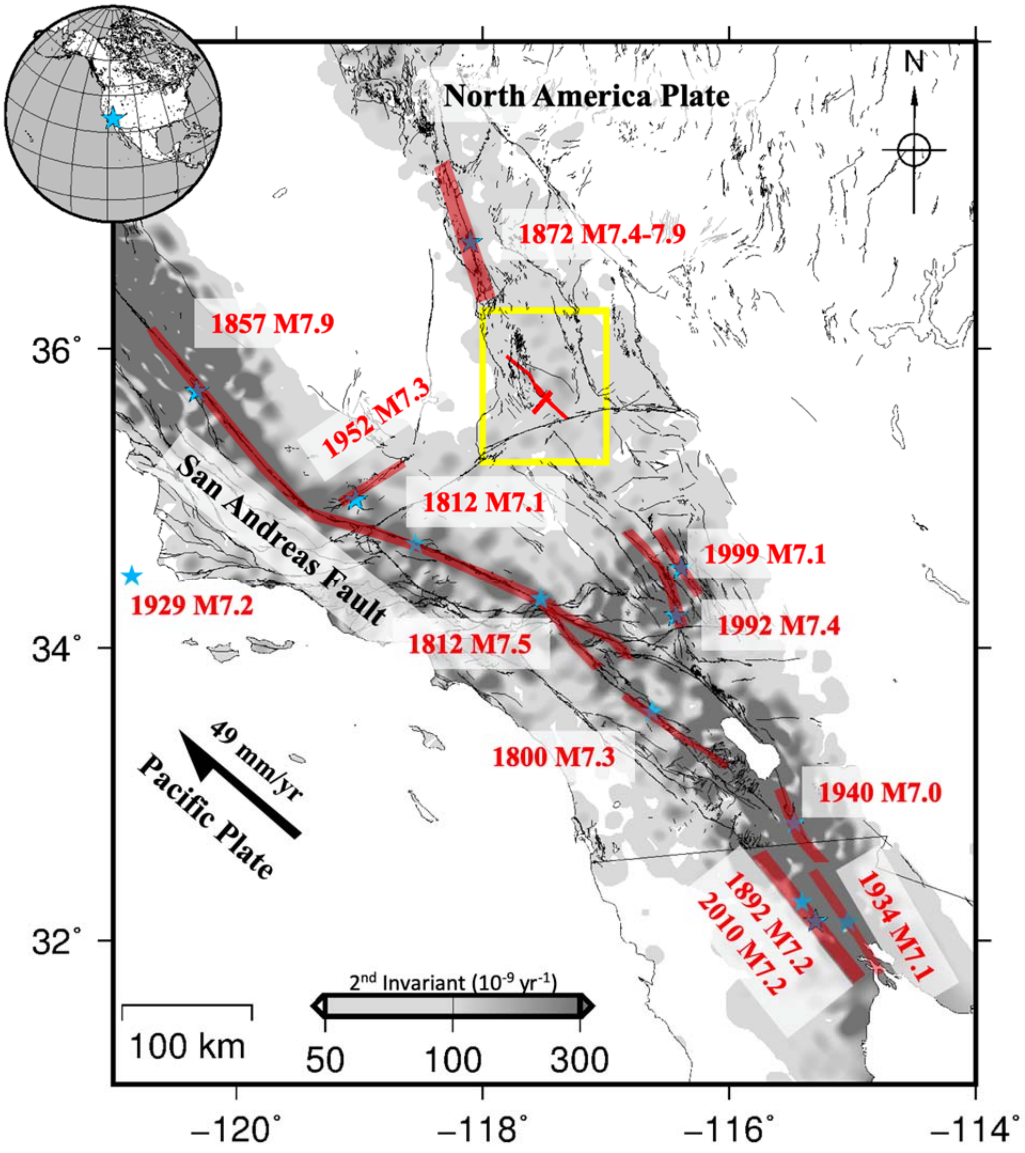

Reviewer #2 (Remarks to the Author):

The authors have addressed all of the issues I have raised, and the manuscript is now suitable for publication.

There is one minor error in new text on L464-465, and I suggest rewording as follows:

"...the coefficient of variation (CV) can be a better measure for showing which features are persistent."

Response: Done.
